# Metal nanomaterials: Immune effects and implications of physicochemical properties on sensitization, elicitation, and exacerbation of allergic disease

**DOI:** 10.1080/1547691X.2019.1605553

**Published:** 2019-12

**Authors:** Katherine A. Roach, Aleksandr B. Stefaniak, Jenny R. Roberts

**Affiliations:** a Allergy and Clinical Immunology Branch (ACIB), National Institute of Occupational Safety and Health (NIOSH), Morgantown, WV, USA;; b School of Pharmacy, West Virginia University, Morgantown, WV, USA;; c Respiratory Health Division (RHD), National Institute of Occupational Safety and Health (NIOSH), Morgantown, WV, USA

**Keywords:** Nanotoxicology, allergy, metal nanoparticles, lung function, immune response

## Abstract

The recent surge in incorporation of metallic and metal oxide nanomaterials into consumer products and their corresponding use in occupational settings have raised concerns over the potential for metals to induce size-specific adverse toxicological effects. Although nano-metals have been shown to induce greater lung injury and inflammation than their larger metal counterparts, their size-related effects on the immune system and allergic disease remain largely unknown. This knowledge gap is particularly concerning since metals are historically recognized as common inducers of allergic contact dermatitis, occupational asthma, and allergic adjuvancy. The investigation into the potential for adverse immune effects following exposure to metal nanomaterials is becoming an area of scientific interest since these characteristically lightweight materials are easily aerosolized and inhaled, and their small size may allow for penetration of the skin, which may promote unique size-specific immune effects with implications for allergic disease. Additionally, alterations in physicochemical properties of metals in the nano-scale greatly influence their interactions with components of biological systems, potentially leading to implications for inducing or exacerbating allergic disease. Although some research has been directed toward addressing these concerns, many aspects of metal nanomaterial-induced immune effects remain unclear. Overall, more scientific knowledge exists in regards to the potential for metal nanomaterials to exacerbate allergic disease than to their potential to induce allergic disease. Furthermore, effects of metal nanomaterial exposure on respiratory allergy have been more thoroughly-characterized than their potential influence on dermal allergy. Current knowledge regarding metal nanomaterials and their potential to induce/ exacerbate dermal and respiratory allergy are summarized in this review. In addition, an examination of several remaining knowledge gaps and considerations for future studies is provided.

## Introduction

Over the past several decades, an extensive amount of scientific attention has been invested in the field of nanotechnology. Significant advances have been made in understanding the unique behaviors of matter with nano-scale dimensions. This progress has facilitated the capacity for manipulation of material properties to optimize their functional utility. Subsequently, nanomaterials have proven useful in diverse applications ranging from pharmaceutics and energetics to transportation and electronics. The exponential growth of the nanotechnology field has left few sectors unaffected by its momentum, as the global nanotechnology market has been valued at over $20 billion US (Nanomaterials Future Markets 2015). Although the resounding impact of these technological advancements has generated comparisons to the impact of the industrial revolution, the expansion of nanotechnology has also generated several notable concerns. In addition to the environmental, legal, ethical, and regulatory challenges imposed by the expanding presence of nanotechnology, the potential risk for adverse health effects following exposure to nanomaterials has also become a major concern.

**Figure 1.**
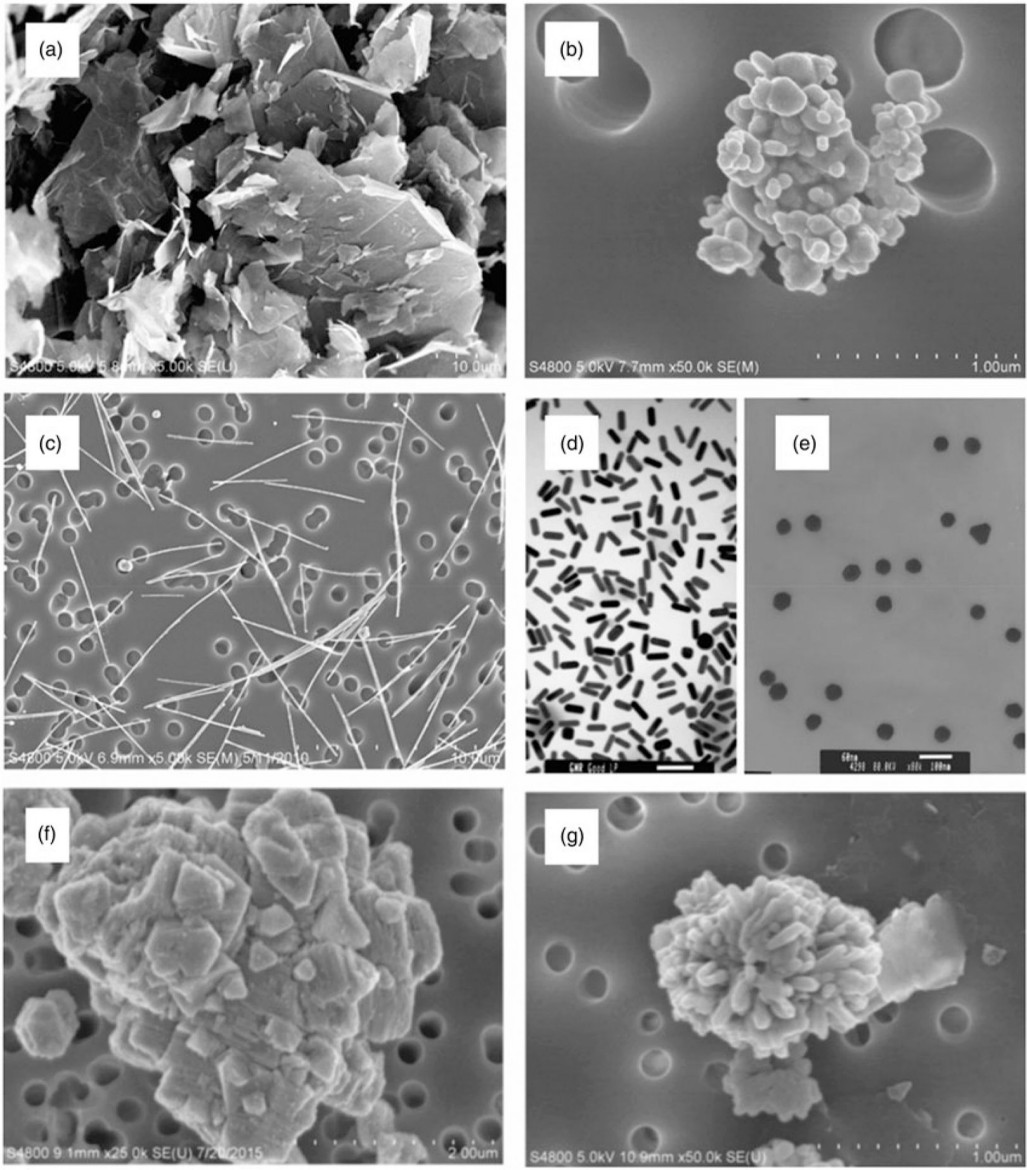
Different morphologies of nanomaterials are shown: (a) graphene sheets, (b) silver nanoparticles, (c) silver nanowires, (d) gold nanorods, (e) gold nanoparticles, (f) nickel oxide nanoparticles, and (g) copper oxide nanoparticles.

As a result, a unique discipline of toxicology has emerged to evaluate the potential health effects of nanomaterials. Nanotoxicology studies have consistently demonstrated that the unique properties of nanomaterials that render their industrial functionality also implicate unique interactions with biological systems. A general correlation between decreasing size and increased toxic potential has been observed for many nanomaterials (Shang et al. 2014). However, additional physical and chemical properties of nanomaterials have been implicated in their biological activity. Nanomaterials exist in various morphologies (Figure 1) with diverse surface textures, and can assume differing degrees of agglomeration. These physical properties contribute to variations in their chemical properties, which include surface charge, dissolution kinetics, and surface reactivity (Oberdorster et al. 2005; Castranova 2011; Gatoo et al. 2014).

One of the greatest challenges presented to nano-toxicologists arises from the discord between the rapid emergence of vast quantities of new nanomaterials and the significant amount of time and resources required to evaluate the safety of each material individually. A novel risk assessment approach proposed to mitigate this issue involves delineation of relationships between specific physicochemical properties and toxicological modes of action (Kuempel et al. 2012; Braakhuis et al. 2016). Subsequently, emerging materials can be categorized by this scheme, providing preliminary safety information and prioritization of resources for *in vivo* studies (Schulte et al. 2014). Significant advancements have been made using this approach with respect to toxic effects on the lungs, but the correlation of nanomaterial physicochemical properties with adverse effects on other systems, such as the immune system, are less clear.

In addition to protecting the host from both endogenous and exogenous threats, the immune system is a critical regulator in hundreds of other disorders, as inflammation is a critical component in the pathophysiology of nearly all chronic diseases states (Pawelec et al. 2014). Accordingly, deviations in optimal immune functioning can have resounding effects on host health, whether polarized towards being either stimulatory or suppressive in nature. One of the immunological disorders presenting a significant and continually expanding global public health burden is allergy. The term “allergic disease” refers to a collective assortment of disorders involving diverse inciting agents, underlying immunological mechanisms, and clinical manifestations. However, all hypersensitivity disorders are characterized by commonality in hyperactivation of adaptive immune responses directed at otherwise innocuous exogenous antigens (Pawankar 2014).

Rates of allergic disease have been on the rise for decades, and the American Academy of Allergy, Asthma, and Immunology reports that worldwide, sensitization rates to one or more common allergens are approaching 40–50% in school-aged children (AAAAI 2015). In the United States, allergic diseases are the sixth leading cause of chronic illness with an annual cost exceeding $18 billion US (Centers for Disease Control and Prevention 2017). Although the development of allergy is dependent on a multitude of genetic, behavioral, and environmental factors, exposures to immunotoxic agents are a major underlying contributor to allergic diseases (Boverhof et al. 2008). Immunotoxic agents with the capacity to impact allergic disorders generally exert one of two effects. First, the agent can act as an allergen or sensitizer. Following exposure to these agents, the resultant adaptive immune response is specific to the agent and subsequent encounters trigger allergic reactions. Contrarily, agents can augment immunological processes involved in allergic disorders specific to differing agent. These agents are often referred to as “adjuvants” or “immuno-modulators” and their effects can range from increasing host susceptibility to sensitization, decreasing the allergen dose required to induce sensitization, decreasing the dose required to elicit allergic responses, or exacerbating the severity of allergic responses (Zunft 1996).

As the nanotechnology market continues to expand and the global prevalence of allergic disease continues to increase, the knowledge gap regarding the immunotoxic potential of nanomaterials is becoming increasingly relevant. Specifically, the capacity for nanomaterials to cause or exacerbate allergic disease remains largely unknown, which is particularly concerning with respect to a specific class of nanomaterials. Metal-based nanomaterials (e.g. metallic, oxidic, alloy, and salt forms) are one of the classes of nanomaterials being produced in the largest quantities. Noteworthy metal nanomaterials, their applications, and corresponding rates of production are shown in Table 1. These emerging materials present a specific concern with respect to allergy, as many of the metal-based nanomaterials being manufactured in large volumes are comprised of metals known to cause allergic contact dermatitis (ACD), asthma, and allergy adjuvancy (Warshaw et al. 2013; Schmidt and Goebeler 2015).

Metal nanomaterials are being increasingly incorporated into nano-enabled products and consumer goods, increasing the potential for exposures in the general public (Vance et al. 2015). Although the unknown immune effects of many metal nanomaterials present a risk for consumers, workers involved in the manufacture, handling, and transportation of metal nanomaterials present a population particularly susceptible to adverse allergic effects. The National Science Foundation estimates that by 2020, at least 2 million workers in the United States alone will be employed by nanotechnology-related fields (Roco 2011). New nanomaterials are continually being developed, and workers are often the first individuals in society to encounter emerging materials, and often in much greater quantities than consumers. Moreover, occupational settings are known to contribute to the development and progression of allergic diseases (Arruda et al. 2005). Of the 24 million Americans affected by allergic asthma, 10–25% of adult cases are related to workplace conditions (Petsonk 2002). Skin allergies are even more common, affecting an estimated 15–20% of the general population, wherein an estimated 25–60% of ACD cases are related to occupational settings (Diepgen and Coenraads 1999; Peiser et al. 2012).

Several specific concerns have emerged with respect to metal nanomaterials and their potential effects on allergic disease. First, the characteristic size profile of metal nanomaterials may confer enhanced potential for skin penetration and more efficient deposition in the lungs, circumventing one of the major barriers associated with limiting adverse immune effects induced by larger-sized metals. Secondly, the exposure threshold required for dermal and respiratory sensitization by metal nanomaterials may be lowered as a result of their unique chemistry. Lastly, the induction of inflammation and tissue injury caused by many metal nanomaterials may serve as an adjuvant, promoting the development of allergic disease to environmental allergens or exacerbating the severity of established allergic conditions. This review aims to summarize current scientific knowledge regarding these concepts. In addition to dermal and respiratory studies that examine specific metal nanomaterial effects, studies designed to delineate the role of physical and chemical properties in these effects are emphasized. Finally, considerations and knowledge gaps in the field are highlighted as potential directions for future research.

## Metals and allergic disease

Metals are a class of agents associated with expansively diverse immune effects including irritancy, autoimmunity, sensitization, and adjuvancy (Lawrence and McCabe 2002). The potential for such diverse biological effects is reflective of the expansive potential speciation of metals, which can include elemental forms, ions, salts, and organified compounds (Templeton 2015). Moreover, as exemplified by the transition metal series, many metals exist in and transition between different oxidation states that have distinctive immunological activity (Artik et al. 1999; Crichton 2017). The chemical behavior unique to these potential states dictates the molecular and cellular interactions responsible for metal immunogenicity. Since many of these properties are known to be altered on the nano-scale, metal-induced immune effects with relevance to allergy are detailed, with emphasis on specific processes subject to impact by metal nanomaterial physicochemical properties.

### Metals and dermal allergy

**Figure 2.**
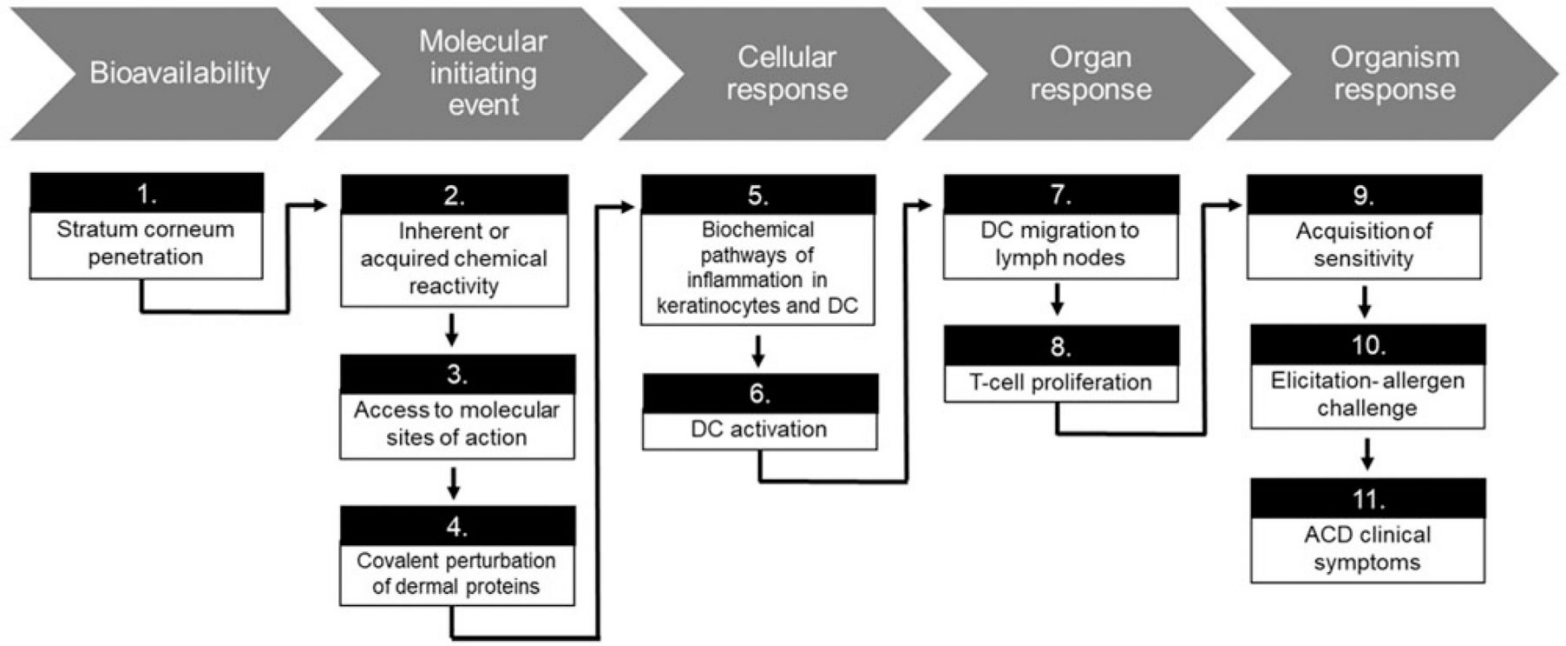
Steps of the Adverse Outcome Pathway (AOP) for dermal sensitization adapted from the Organization for Economic Cooperation and Development (OECD).

The most common metal-induced allergic disorder of the skin is ACD, a T-cell-mediated delayed-type hypersensitivity response. Dermal sensitization and the subsequent induction of ACD requires several key molecular and cellular events (Figure 2), which have been outlined in an adverse outcome pathway (AOP) by the Organization for Economic Co-operation and Development (OECD) (OECD 2014).

The preliminary requirement for skin sensitization is bioavailability of the sensitizing agent. Since a primary function of the skin is to serve as an effective barrier between the host and environment, the sensitizing potential of many antigens is limited by their capacity to evade this barrier (Jaitley and Saraswathi 2012). Passage through the uppermost layers of the epidermis is heavily dependent on antigen physical and chemical properties. Likewise, most dermal sensitizers tend to be low molecular weight (LMW, < 500 Daltons) chemicals with adequate lipophilicity (logP ~2) (Chilcott and Price 2008; Karlberg et al. 2008). Metals associated with skin sensitization present the greatest concern when formulated as soluble salts that release ions capable of penetrating the physical barrier presented by the epidermis (Di Gioacchino et al. 2007; Kubo et al. 2013).

The next steps in the skin sensitization AOP involve the molecular initiating event of skin sensitization-antigen formation. The small size required for antigen passage through the stratum corneum is not conducive with cellular recognition (Anderson et al. 2011). As a result, most skin sensitizers are referred to as haptens, which must acquire or possess inherent chemical reactivity that facilitates binding to carrier molecules (Büdinger et al. 2000; Chipinda et al. 2011). This process generates adequate size for recognition by an antigen-presenting cell (APC). The APC most frequently implicated in dermal sensitization is the resident dendritic cell (DC) of the epidermis, the Langerhans cell (LC) (Thyssen and Menne 2010).

In addition to uptake of the hapten/carrier complex, activation of LC requires an additional antigen-nonspecific signal indicative of an elevated threat level. Many mediators capable of fulfilling this signal are released by non-immune cells including keratinocytes in response to injury (Dearman and Kimber 2003). Presence of both antigen-specific and nonspecific signals induce LC maturation, upregulation of co-stimulatory molecules, antigen processing, and migration to the lymph nodes (Tončić et al. 2011).

Once the LC reaches the lymph nodes, the processed hapten is presented via major histocompatibility Class I (MHC I) molecules to naïve CD8^+^ T-lymphocytes until recognition occurs by antigen-specific T-cell receptor (TCR). Given adequate costimulatory signals from the LC, T-lymphocytes undergo proliferation producing a pool of clonal antigen-specific effector T-cells. The T-cells enter the circulation, and following resolution of inflammation, a subset of these effector cells will survive and become memory T-cells, completing the process of sensitization.

Upon future exposures to the allergen, memory and effector T-cells are recruited to the site of exposure where CD8^+^ T-cells exhibit immediate cytotoxic effector functions. CD4^+^ T-helper (T_H_) of the T_H_1 phenotype have regulatory roles in ACD and produce high levels of the cytokines interleukin (IL)-2 and interferon (IFN)-γ, contributing to inflammatory cell recruitment (Sasseville 2008; Tončić et al. 2011). Within 48 h, the inflammatory process originally orchestrated to destroy the antigen results in the clinical manifestations of ACD, including localized skin redness, swelling, and itching at the site of allergen contact.

Metals are among the most common inducers of ACD in the general population. Patch test studies have generated data from thousands of subjects and reveal that the most common inducers of metal ACD are nickel, gold, cobalt, and chromium (Belloni Fortina et al. 2015). Interestingly, studies using subjects exclusive various geographical locations have demonstrated that these four metals are consistently problematic with respect to ACD worldwide (Kanerva et al. 2000; Mattila et al. 2001; Goon and Goh 2005; Cheng et al. 2008; Davis et al. 2011; Nonaka et al. 2011; Khatami et al. 2013; Mahler et al. 2014; Kim et al. 2015; Malinauskiene et al. 2015; Linauskiene et al. 2017). Though less frequently associated with ACD, copper, aluminum, and platinum group metals are also known to cause skin allergy in some individuals (Hostynek et al. 1993; Bergfors et al. 2005; Faurschou et al. 2011; Fage et al. 2014).

### Metals and respiratory allergy

Metals are also associated with respiratory allergy and IgE-mediated asthma. An AOP specific to the events of respiratory sensitization has not been adopted by the OECD, but many of the same steps of the dermal sensitization AOP are involved in the development of asthma. Accordingly, bioavailability of the sensitizing agent is also a preliminary limiting factor in respiratory sensitization potential. Since the primary function of the respiratory tract is to facilitate gas exchange between the host and environment, it is particularly vulnerable to adverse effects from a diverse assortment of agents in the inhalable (< 20 μm) and respirable size range (< 10 μm) (Elder and Oberdorster 2006). Likewise, bioavailable metals capable of sensitizing the respiratory tract are not limited to ions, like in the skin (Linde et al. 2017). Respirable metals may be encountered as particulate matter, vapors, or fumes and can be constituents of compounds including oxides, sulfides, and salts, or as complexes with ammonia, carbon monoxide, and organic nitrogen (Malo et al. 2013).

Since both low and high molecular weight (HMW) agents are capable of inducing asthma, the molecular initiating events of respiratory sensitization may differ accordingly. Similar to the skin, pulmonary immune responses following inhalation of metals can be nonspecific and self-limiting, or can result in the recruitment of the adaptive immune system. Lung-resident DC take up antigen, and given adequate second signals, the antigen is processed and DC migrate to the lung-draining lymph nodes. Here, the peptide is presented to naïve CD4^+^ T-lymphocytes along with costimulatory molecules, resulting in the preferential expansion of the T_H_^−2^ phenotype lymphocytes. These cells produce high levels of IL-4, IL-5, and IL-13, and stimulate isotype switching and allergen-specific IgE-production by B-cells. The Fc portion of secreted IgE is bound to FceRI receptors present on tissue-resident mast cell surfaces and circulating basophils, exposing the antigen-recognizing motif, completing the sensitization process (Verstraelen et al. 2008).

Upon subsequent encounters, the allergen is bound by allergen-specific IgE on the surface of mast cells and basophils. Binding induces crosslinking of receptors and the subsequent release of preformed mediators such as histamine, beginning the anaphylactic cascade responsible for the early asthmatic reaction experienced minutes after antigen encounter. Acute clinical manifestations of allergic asthma range from rhinitis and bronchoconstriction to anaphylactic shock. The late phase asthmatic response occurs 4–6 h later as a result of mast cell mediators and recruitment of inflammatory cells (Possa et al. 2013). Clinical presentations of the late phase asthmatic response tend to be more severe than early phase responses, and include excessive mucus production, increased vascular permeability, and airway constriction. Chronic cycles of allergic inflammation and subsequent repair are associated with structural alterations in the airways that can have physiological implications, such as a decline in lung function (Erle and Sheppard 2014).

Compared to metal-induced ACD, metal-induced asthma occurs far less frequently. Cases tend to be isolated to individuals working in occupations involving metalwork where metal fumes, dust, or vapors are generated and inhaled (Wyman and Hines 2018). Nickel, chromium, cobalt, vanadium, zinc, platinum and aluminum have all been associated with cases of occupational asthma (Musk and Tees 1982; Hong et al. 1986; Malo et al. 2013). However, metal-specific IgE has only been implicated in cases caused by nickel, platinum, chromium, and cobalt (Malo et al. 1982; Murdoch et al. 1986; Shirakawa et al. 1988, 1990, 1992; Kusaka et al. 1996). Metal-specific IgG molecules have also been implicated in cases of cobalt and platinum-induced asthma (Pepys et al. 1979; Cirla 1994). The presence of metal-specific IgE has also been confirmed in the absence of asthmatic symptoms, emphasizing the potential for numerous immunological mechanisms in metal-specific asthma (El-Zein et al. 2005; Tončić et al. 2013).

### Metals and allergy adjuvancy

**Figure 3.**
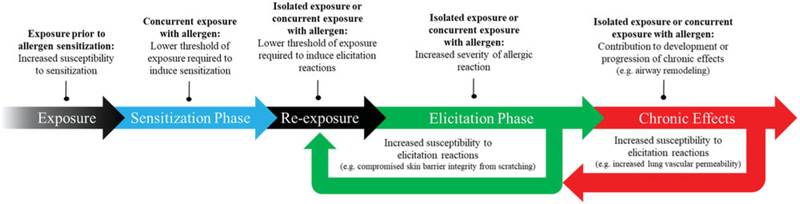
Potential adverse outcomes with respect to the sensitization and elicitation phases of allergy following exposure to immunotoxic agents. Adjuvant effects resulting from exposure prior to allergen sensitization can manifest as increased susceptibility to sensitization. Exposure concurrent to sensitization may lower the threshold of allergen exposure required to induce sensitization. Following sensitization to allergen, exposure to an immunotoxic agent either in the absence or presence of allergen may result in a lower threshold of exposure required to induce elicitation reactions or increased severity of elicitation symptoms. These effects may further increase susceptibility to elicitation reactions as result of physiological alterations such as compromised skin barrier integrity. Furthermore, isolated exposure to immunotoxic agents or concurrent to allergens in established allergic disease conditions may also contribute to the progression of chronic effects, such as airway remodeling, which can also further contribute to elicitation reactions.

In addition to their potential to induce sensitization, metals are also associated with the capacity to modulate allergic responses to nonmetal allergens. Contrasted with the consistent, sequential series of cellular processes involved in allergic sensitization and elicitation, adjuvant effects can emerge as a result of various mechanisms in various phases of allergic disorders (Figure 3).

An example of metal adjuvant effects on the development of adaptive immune responses is best demonstrated by aluminum hydroxide, which is one of the most frequently used vaccine adjuvants. When administered with poorly-immunogenic antigens, aluminum hydroxide induces adequate stimulation of the innate immune system to generate antigen-specific immunological memory. Mechanisms of immunopotentiation associated with aluminum hydroxide include triggering release of alarmins, activation of inflammasomes (intracellular multi-protein complexes involved in innate immune responses), and DC activation; however, numerous other mechanisms including immune cell recruitment and activation, modulated cytokine production, and altered antigen delivery kinetics, can also enhance sensitization (Naim et al. 1997; Aimanianda et al. 2009).

Similarly, metals are also associated with adjuvant effects on established allergic conditions, as demonstrated by metal-rich ambient air pollution, which is known to exacerbate the severity of asthmatic responses to environmental allergens (Gavett et al. 2003; Schaumann et al. 2004). Adjuvant effects on allergic elicitation can involve mechanisms including induction of pulmonary oxidative stress, enhanced degranulation of mast cells, and recruitment of inflammatory cells (Walczak-Drzewiecka et al. 2003; Ghio 2008).

### Unique properties associated with metal immune effects

Metals are known to induce many unique immune effects implicated in allergic disease. With respect to sensitization, the modulation of innate immune reactivity by some metals has been associated with their immuno-genicity. For example, some metal ions are known to produce functional mimicry of pathogen-associated molecular patterns (PAMP) (Schmidt and Goebeler 2015). Gold ions have the capacity to bind and activate Toll-like receptor (TLR)-3, while nickel, cobalt, and palladium ions have the capacity to bind and activate human TLR-4 (Schmidt et al. 2010; Rachmawati et al. 2013, 2015). The subsequent induction of pro-inflammatory signaling generates the antigen nonspecific signals required for DC activation, promoting sensitization.

Metals are also known to modulate mechanisms of communication between innate and adaptive immune cells. Accordingly, antigen presentation is another step in allergic sensitization that is subject to interference by metals. Beryllium and noble metals have been shown to induce structural alterations in MHC molecules, impacting subsequent interactions with TCR (de Wall et al. 2006; Falta et al. 2013). Similarly, peptide-independent linking of MHC and TCR by nickel has been demonstrated (Gamerdinger et al. 2003; Thierse et al. 2005). Adaptive immune responses can also be affected by metals, as demonstrated by CD4^+^ nickel-specific T-cell clones, which were shown to cross-react when presented with other transition metals including copper and palladium (Moulon et al. 1995; Pistoor et al. 1995).

Many of the unique immune effects associated with metals emerge as a result of their capacity to alter molecular and cellular interactions on a biochemical level. Accordingly, their modulation of processes involved in allergic disease is critically dependent on physicochemical properties including special geometry, oxidation state, and solubility (Schuhmann et al. 1990; Kinbara et al. 2011). Many of these properties are altered in nanoparticulate form, suggesting that metal nanomaterials may exhibit novel mechanisms of immune interaction with implications for allergic disease.

## Metal nanomaterials and dermal hypersensitivity

The potential for adverse immune effects following dermal exposure to metal nanomaterials is a growing concern due to their increasingly frequent incorporation into consumer goods intended to have prolonged contact with the skin (Yang and Westerhoff 2014). The unique optical properties of titanium dioxide nanoparticles (TiO_2_ NP) and zinc oxide nanoparticles (ZnO NP) have led to their incorporation in sunscreens and cosmetics for their protective effects against ultraviolet radiation (UVR) (Smijs and Pavel 2011; Yoshioka et al. 2017). Silver nano-particles (AgNP) are being incorporated into clothes, medical textiles, toys, and cleaning products due to their antimicrobial properties, and silica-based nanoparticles (SiNP) have been frequently used in cosmetics and as a coating material to alter the properties of other materials (Contado 2015; Tulve et al. 2015). Likewise, the dermal effects of TiO_2_ NP, ZnO NP, AgNP, and SiNP are a particular concern with respect to the general public (Weir et al. 2012). These nanomaterials are also a concern for workers, but other metal nanomaterials with high rates of production (listed in Table 1) are also associated with dermal exposures in the workplace.

The potential for metal nanomaterials to penetrate the skin, induce dermal sensitization, and modulate skin allergy development/responses are the three main areas discussed in this section with respect to size and other physicochemical properties. In correspondence with the review of the literature, Table 2 summarizes studies characterizing effects of individual metal nanomaterials on skin allergy. Table 3 summarizes studies designed to examine effects of physicochemical properties of metal nanomaterials on dermal allergy. Table 4 highlights key events involved in dermal sensitization and elicitation that have been shown to be subject to modulation by metal nanomaterials and their corresponding physicochemical properties.

### Skin penetration and translocation studies

Adverse immune effects following dermal exposure to an agent are heavily dependent on the degree to which the skin protects from its entry into the body. Likewise, one mechanism by which dermal exposure to metal nanomaterials may lead to increased potential for adverse immune effects compared to larger-sized metals is by size-mediated evasion of skin barrier function. Although it seems logical that the small size of nanomaterials would inherently provide increased opportunity for absorption via the skin, there is currently no general consensus on the skin-penetrating capabilities of nanomaterials as a collective class of agents (Vogt et al. 2006; Baroli 2010; Try et al. 2016).

Numerous studies have demonstrated that metal nanomaterials (< 100 nm) can penetrate skin in various *in vivo* and *in vitro* models. Iron-based nanoparticles (FeNP), gold nanoparticles (AuNP), palladium nanoparticles (PdNP), nickel nanoparticles (NiNP), AgNP, SiNP, and metal-based quantum dots (QD) have all been associated with penetration of the skin (Baroli et al. 2007; Chu et al. 2007; Filon et al. 2011, 2016; Labouta et al. 2011; Hirai et al. 2012a; Rancan et al. 2012; George et al. 2014; Crosera et al. 2016; Kraeling et al. 2018). Moreover, many of these studies have established a relationship between decreased particle size and increased potential for skin permeation (Ryman-Rasmussen et al. 2006; Sonavane et al. 2008; Matsuo et al. 2016; Raju et al. 2018). Hydrophobicity, surface charge, and morphology are additional properties that have been shown to be influential in the capacity for these nanomaterials to pass through the stratum corneum (Rancan et al. 2012; Lee et al. 2013; Iannuccelli et al. 2014; Fernandes et al. 2015; Tak et al. 2015; Mahmoud et al. 2018).

By comparison, the majority of studies investigating the skin-penetrating potential of metal nanomaterials have been conducted with ZnO NP and TiO_2_ NP and have not generated equally consistent findings. Numerous studies have demonstrated that the stratum corneum effectively restricts passage of TiO_2_ NP, irrespective of size shown to facilitate penetration of the skin by other metal nanomaterials. Repeated application of different forms of TiO_2_ NP did not lead to skin penetration in hairless rats, elevated levels of titanium in lymph nodes of minipigs, or penetration of human skin transplanted onto immunodeficient mice (Kiss et al. 2008; Sadrieh et al. 2010; Adachi et al. 2013). Although TiO_2_ NP were shown to accumulate in and around furrows of the skin, microscopic analysis was used to confirm that 20–100 nm TiO_2_ NP remained restricted to the uppermost 3–5 layers of corneocytes of the stratum corneum (Lademann et al. 1999; Pflücker et al. 1999; Gontier et al. 2008; Senzui et al. 2010). Contrarily, a few studies using TiO_2_ NP-containing sunscreens have reported penetration of particles into the viable epidermis of human skin (Tan et al. 1996; Coelho et al. 2016; Naess et al. 2016).

Similar observations have been reported for ZnO NP. Despite associations with hair follicles, ZnO NP were not capable of penetrating the stratum corneum in multiple models, irrespective of alterations in size, morphology, and surface characteristics (Schulz et al. 2002; Zvyagin et al. 2008; Leite-Silva et al. 2013). However, ion release from ZnO NP and ZnO NP-containing sunscreens has been observed, highlighting a potential risk associated with soluble metal nanomaterials (Holmes et al. 2016).

Adverse effects following dermal exposure to nanomaterials may result from penetration of the particulate material or ions released from the parent material. Likewise, physicochemical properties of interest may be differentially implicated in effects associated with soluble and insoluble metal nanomaterials. With respect to soluble materials, properties associated with accelerated ion release may indicate increased potential for skin penetration (Lansdown 1995; Hostynek 2003; Thyssen and Menne 2010). The rate of ion release is proportional to specific surface area (SSA; surface area per mass unit), which is exponentially increased on the nano-scale (Laudańska et al. 2002; Zhang et al. 2011; Larese Filon et al. 2015). This concept explains the observation that application of sunscreens containing ZnO NP caused greater increases in blood, urine, and organ Zn ion levels than sunscreens containing larger-sized ZnO particles (Gulson et al. 2010, 2012; Osmond-McLeod et al. 2014). Moreover, the manipulation of properties with implications for dissolution potential, such as particle coating, vehicle, and suspension pH have been shown to promote Zn ion release from ZnO NP following dermal exposure (Leite-Silva et al. 2013; Holmes et al. 2016).

Although penetration through corneocytes of the stratum corneum is the primary pathway associated with skin penetration by materials, appendages including hair follicles, sebaceous glands, sweat glands, and skin folds can mediate an additional mechanism of skin penetration. This pathway has notable relevance to nanomaterials, as evidenced by the utility of the trans-follicular delivery route for nano-scale pharmaceutics and vaccines (Mahe et al. 2009). Compared to the thickness of the stratum corneum, which measures 10–20 mm, hair follicles can reach a tissue depth of 2000 mm (Toll et al. 2004). Since the base of hair follicles extends into the dermis and receives generous lymph and blood supply, they may promote access into the circulation (Elder et al. 2009). Moreover, hair follicles can serve as a potential reservoir, promoting accumulation of nanomaterials. Retention in hair follicles can extend the duration of exposure 10-fold, raising specific concerns for continual ion release (Lademann et al. 2006, 2009, 2015; Patzelt et al. 2011; Mahmoud et al. 2017). This pathway of skin penetration may also favor immune responses since hair follicles are surrounded by dense networks of LC and specialized keratinocyte subpopulations known to have critical roles in the early events of sensitization (Toews et al. 1980; Vogt et al. 2006; Nagao et al. 2012).

The diameter of hair follicles can vary greatly in response to anatomical location, but the smallest follicles tend to be located on the forehead and forearm and measure between 66 and 78 mm (Otberg et al. 2004). Interestingly, the optimal size for penetration of hair follicles is significantly larger than the < 100 nm size range associated with increased skin penetration of several metal nanomaterials. Particles with 600–700 nm diameter have been shown to deposit in the deepest depths of hair follicles, suggesting that agglomerates of nanomaterials in this size range are potentially more hazardous than primary particles (Patzelt et al. 2011; Lademann et al. 2015). Furthermore, preferential accumulation in follicles has been observed in hydrophobic and neutrally-charged nanomaterials (Mahmoud et al. 2017).

Although physicochemical properties of metal nanomaterials have been shown in some instances to impact skin penetration, an assortment of host factors can also impact this process. Variations in epidermal thickness, integrity, degree of hydration, and skin pH, all of which may further differ between gender, can greatly influence skin permeability (Sandby-Moller et al. 2003; Senzui et al. 2010; de Matteis et al. 2016). However, the role of disrupted skin barrier integrity is one of the most commonly-examined host factors with applicability to allergic disease since skin permeability can be increased 4–100 times in individuals with skin allergy (Larese Filon et al. 2016).

Scratching to alleviate itching associated with allergic skin lesions leads to mechanical damage to the upper layers of skin. Similar degrees of damage have been shown to increase *in vivo* penetration of some metal nanomaterials in humans and rodents (Zhang and Monteiro-Riviere 2008; Gopee et al. 2009; Ravichandran et al. 2011; Prow et al. 2012). *In vitro* simulations using a human skin model called the Franz Method have demonstrated increased capacity for passage through damaged skin by 25 nm AgNP, 6 nm PtNP, 5 nm rhodium nanoparticles (RhNP), 10 nm PdNP, 78 nm NiNP, and 80 nm CoNP (Larese et al. 2009; Larese Filon et al. 2013; Mauro et al. 2015; Crosera et al. 2016; Filon et al. 2016). Contrarily, studies have shown that penetration of various sizes of TiO_2_ NP and ZnO NP are not increased in skin damaged by chemical irritants, tape-strip-ping, hair removal, or mechanical force (Senzui et al. 2010; Lin et al. 2011; Miquel-Jeanjean et al. 2012; Crosera et al. 2015; Xie et al. 2015; Leite-Silva et al. 2016).

A few *in vivo* studies have also investigated effects of skin barrier dysfunction resulting from existing skin allergy on the penetration of metal nanomaterials (Larese Filon et al. 2016). In a mouse model of skin allergy, ZnO NP skin penetration of allergic skin was size-dependently increased, as 240 nm ZnO particles did not penetrate the skin to a similar degree as 20 nm ZnO NP (Ilves et al. 2014). Studies using nonmetal nanomaterials have also demonstrated that penetration of nanomaterials in allergic skin is size-dependent (Try et al. 2016). ZnO NP were also shown to penetrate allergic skin *ex vivo* using human skin samples (Szikszai et al. 2011). Contrarily, application of 35 nm ZnO NP to skin of living human subjects with atopic dermatitis did not lead to penetration into viable skin (Lin et al. 2011). Discrepancies between these studies may be reflective of varying exposure durations, as the study reporting penetration involved continuous exposure of up to 2 weeks, compared to the 4-hr exposure wherein no penetration was observed.

Comparatively, equally prolonged exposure to AgNP-containing textiles did not lead to increased skin penetration in individuals suffering from atopic dermatitis compared to control subjects. Sleeves containing silver particles (30–500 nm) were worn by human subjects for 8 h a day for 5 consecutive days, following which levels of AgNP and aggregates in the skin were quantified. Compromised skin barrier was not associated with increases in AgNP skin accumulation; moreover, no differences in urine Ag levels were observed, indicating that atopic dermatitis did not impact the absorption of ions released from the textiles either (Pluut et al. 2015; Bianco et al. 2016).

Discrepancies in findings regarding the importance of skin barrier integrity on metal nanomaterial skin penetration may be explained by several observations. The diverse degrees of epidermal barrier function disruption between studies represent a potential source of variation. Complete ablation of epidermal function is only observed in response to severe burns and lacerations; likewise, the diverse mechanisms of experimentally-induced disruptions of the stratum corneum should be compared cautiously. In addition, the pathogenesis of atopic dermatitis between experimentally-induced animal models and humans may explain some discordant findings. The severity of lesions between subjects of human studies is also subject to extreme variation, as well. Since chronic skin inflammation can result in epidermal thickening, enhanced barrier function is not uncommon in many skin disorders (Nohynek et al. 2007). Lastly, differences in exposure conditions and duration, test material formulation, and method of penetration assessment can also serve as a source of variation in conclusions between studies. A notable distinction should be made between test materials, since some studies used pristine metal nanomaterials, and others used commercially-available TiO_2_ NP/ZnO NP-containing sunscreens. As noted by Gulson et al. (2012), their observations regarding ZnO NP skin penetration may have been subject to modulation by excipients of the commercial sunscreens used in their study. The sunscreen contained isopropyl myristate, a chemical known to enhance the permeability of the skin, as well as EDTA, a chelating agent which may have influenced the release of ions from ZnONP.

In addition to nanomaterial properties and host factors known to influence the capacity for metal nanomaterials to penetrate the skin, environmental factors may also impact this process. One environmental factor with particular relevance to metal nanomaterials and their use in sunscreens is UVR. Although high levels of UV exposure and subsequent sunburn can significantly disrupt epidermal barrier function, low doses of UV exposure are also known to compromise the integrity of the epidermis (Wolf et al. 1993; Holleran et al. 1997; Biniek et al. 2012). Accordingly, several studies have shown that UV exposure prior to topical application of nanomaterials results in greater depth of penetration by ZnO NP, TiO_2_ NP, and QD (Mortensen et al. 2008; Monteiro-Riviere et al. 2011; Mortensen et al. 2013). Moreover, UVR can induce alterations in physicochemical properties of metal nanomaterials that may facilitate their passage through the stratum corneum, such as agglomerate disaggregation and ion release (Martorano et al. 2010; Bennett et al. 2012; Zhou et al. 2012; Ma et al. 2014).

Simultaneously, UVR-induced photoactivation of some metal nanomaterials can facilitate their penetration of the skin. *In vitro*, UVR-induced ROS production by TiO_2_ NP, QD, and ZnO NP has been associated with DNA damage, lipid peroxidation, and mitochondrial permeability in skin cells (Tiano et al. 2010; Wang et al. 2013; Petersen et al. 2014; Mortensen et al. 2015; Xue et al. 2015, 2016). Subsequent cytotoxicity to dermal fibroblasts, keratinocytes, and melanocytes is another mechanism by which skin barrier integrity can become compromised as a result of UVR. *In vivo*, UVR-induced photoactivation of TiO_2_ NP has been associated with increased adherence to the skin, structural rearrangement of the lipid bilayer, and facilitation of large molecule transdermal penetration (Bennett et al. 2012; Turci et al. 2013; Peira et al. 2014; Pal, Alam, Chauhan, et al. 2016; Pal, Alam, Mittal, et al. 2016). Since the degree of ROS produced in response to UVR has been associated with nanoparticle surface area and reactivity, other related properties such as size, degree of agglom-eration, and surface modification may also contribute to skin penetration following UVR exposure (Shen B et al. 2006; Jassby et al. 2012; Yin et al. 2012; Xiong et al. 2013).

Although UVR may contribute to adverse effects following dermal exposure to metal nanomaterials by facilitating skin penetration, it may also present a unique concern with respect to allergy. Many signaling pathways and pro-inflammatory mediators involved in sensitization have been associated with UVR-dependent photoactivation of metal nanomaterials (Murray et al. 2013; Rancan et al. 2014). Moreover, UVR is known to modulate the immune status of the skin by a number of mechanisms. For example, UVA and UVB are known to augment costimulatory molecule expression, compromise antigen presentation, and induce apoptosis of LC (Rattis et al. 1998; Seite et al. 2003; Schwarz 2005). Subsequent effects on the immunological fate of metal nanomaterials on the skin have been demonstrated. In a mouse model, significant depletion of LC (~80%) following UVR exposure increased skin penetration of QD, but resulted in lower levels of metal ion constituents in the lymph nodes (Mortensen et al. 2013).

### Skin sensitization studies

The skin sensitizing potential of metal nanomaterials has been investigated in a few studies using traditional *in vivo* approaches. SiO_2_ NP, ZnO NP, and TiO_2_ NP have all been incorporated into the Local Lymph Node Assay (LLNA) (Mandervelt et al. 1997; Basketter et al. 1999). Accordingly, it was demonstrated that topical exposure to 100 nm mesoporous and colloidal SiO_2_ NP, 7 nm SiO_2_ NP, and ZnO NP were not capable of inducing the 3-fold increase in lymphocyte proliferation associated with classification as a dermal sensitizer (Choi et al. 2011; Lee et al. 2011; Kim et al. 2016). Similarly, topical exposure to 25 nm TiO_2_ NP did not induce dermal sensitization in multiple studies; however, subcutaneous injection of equal doses resulted in significant increases in lymphocyte proliferation, suggesting that the inability for TiO_2_ NP to penetrate the skin might be a limiting factor in the potential to induce dermal sensitization (Park et al. 2011; Auttachoat et al. 2014).

The guinea pig maximization test (GPMT) is another *in vivo* technique used to evaluate dermal sensitization potential that has been employed in the investigation of several metal-based nanomaterials. In one study, five UV-absorbing materials containing SiO_2_ NP, ZnO NP, and TiO_2_ NP were assessed. One out of 10 animals exhibited slight erythema following topical exposure to the ZnO NP and TiO_2_ NP-containing agents, leading to their classifications as mild skin sensitizers (Piasecka-Zelga et al. 2015). In another study, 1 of 20 animals exhibited discrete patchy erythema following intradermal injection with 10 nm AgNP, leading to its classification as a weak skin sensitizer (Kim et al. 2013). Similarly, AgNP were classified as a Grade II (mild sensitizer) after 2 of 10 guinea pigs exhibited lesions 48 h after application of AgNP-containing sterile gauze (Zelga et al. 2016). However, similar AgNP-containing dressings were actually shown to improve the healing of burn wounds in rats over an 18-d period as compared to rats with dressings lacking AgNP, but the study only examined the localized effects (Pannerselvam et al. 2017). The GPMT has also been used to demonstrate that surface-modified FeNP and hydroxyapatite nanoparticles did not induce skin sensitization (Geetha et al. 2013; Mohanan et al. 2014).

The sensitization potential of 5 and 10 nm AgNP was investigated by Hirai et al. (2016) in a mouse model. Mice were injected with AgNP or Ag ions and lipopolysaccharide (LPS) once a week for 4 weeks, and then intradermally challenged. Interestingly, mice administered Ag ions in the sensitization phase did not develop ear swelling following challenge with any form of Ag. Contrarily, AgNP exposure induced sensitization, wherein the smaller AgNP appeared to have stronger sensitizing potential, which was dependent on CD4^+^ T-cells and IL-17a, but not IFNγ. Moreover, ear swelling was observed in response to additional sizes of AgNP (50 and 100 nm) and Ag ions, suggesting that the immune response is not nanoparticle-specific. Further examination revealed that 3 nm NiNP was also capable of inducing sensitization in the model, whereas minimally-ionizable 10 nm AuNP and 10 nm SiNP were not (Hirai et al. 2016).

In addition to *in vivo* approaches to assess skin sensitization, three non-animal alterative assessment methods based on different steps of the skin sensitization AOP are currently validated by the OECD. While metal nanomaterials have not been incorporated into any of the assays, studies with similar cell lines and endpoints have indicated that many metal nano-materials can induce effects similar to those of other skin sensitizers.

The Direct Peptide Reactivity Assay (DRPA) is an *in chemico* assay based on the requirement for haptens to bind skin proteins to acquire immunogenicity. Accordingly, the molecular imitating event of dermal sensitization is evaluated by quantification of reactivity of an agent towards synthetic lysine and cysteine residues (Gerberick et al. 2004). While some studies have investigated metal nanomaterials and their interactions with proteins and specific amino acids, implications for their capacity to form hapten/carrier complexes are still unclear. However, cysteine has been associated with decreased stability, increased dissolution, and accelerated ion release from metal alloy nanoparticles and AgNP (Hahn et al. 2012; Ravindran et al. 2013; Siriwardana et al. 2015). Moreover, various amino acids have been associated with preferential binding affinities with respect to AuNP size and TiO_2_ NP surface charge, supporting a role for multiple physicochemical properties in the molecular initiating event of skin sensitization (Liu et al. 2016; Shao and Hall 2016).

The second validated *in vitro* assay for determination of skin sensitizing potential involves evaluation of the keratinocyte response to test agents, since they are a source of numerous mediators that facilitate LC migration, antigen presentation, and T-cell activation during sensitization (Kimber and Cumberbatch 1992). Since many of these mediators are released in response to sensitizer-induced activation of the antioxidant/electrophile sensing pathway Keap1/Nrf2/ARE, its activation is suggestive of potential for the test agent to contribute to the cellular response of the sensitization AOP (Natsch and Emter 2008; 2016; Ramirez et al. 2014). The human keratinocyte cell line associated with this assay, HaCaT, has been frequently used to investigate potential metal nanomaterial effects on the skin *in vitro*. Correspondingly, PdNP, AuNP, and PtNP have all been shown to activate the Nrf2 pathway in keratinocytes *in vitro* (Goldstein et al. 2016; Tsuji et al. 2017). Similarly, zinc-containing QD, ZnO NP, and CuO NP have all been shown to alter expression of several specific genes associated with the Nrf2 pathway, including *HMOX1* (Rice et al. 2009; Romoser et al. 2011; Lee et al. 2012).

Prior to the establishment of Nrf2 pathway involvement in keratinocyte responses to skin sensitizers, cytokine release by keratinocytes *in vitro* was evaluated as an indicator of sensitizing potential (Jung et al. 2016; Koppes et al. 2017). Tumor necrosis factor (TNF)-α is a keratinocyte-derived cytokine involved in sensitization and is critically involved in skin sensitization by chromium and nickel (Lisby et al. 1995; Wang et al. 2007). Dose-dependent TNFα release has been noted following keratinocyte exposure to AgNP, QD, and ZnO NP, indicating high doses may promote LC maturation and dermal sensitization (Samberg et al. 2010; Romoser et al. 2011; Jeong et al. 2013). IL-18 and IL-1β (cytokines critical for LC activity) have also been shown to be increased by QD, SiO_2_ NP, TiO_2_ NP, and AgNP (Ryman-Rasmussen et al. 2007; Samberg et al. 2010; Yazdi et al. 2010; Romoser et al. 2011; Hiroike et al. 2013; Zhang and Monteiro-Riviere 2019).

Another mediator involved in skin sensitization that is differentially-released by keratino-cytes in response to irritants and sensitizers is IL-1α (Coquette et al. 2003; Koppes et al. 2017). Though it can also be actively secreted after inflammasome activation, IL-1α is an intracellular molecule that functions as an alarmin (Ansel et al. 1988). During programed cell-death, IL-1α remains associated with chromatin and its sequestration prevents effector functions. Contrarily, under necrotic conditions, it is passively released and bioactive. Accordingly, the mechanism of metal nanomaterial-induced keratinocyte cytotoxicity may significantly impact the development of ACD as a result of differential IL-1α release. Although mechanisms associated the preferential induction of necrosis or apoptosis by nanomaterials have yet to be established, some properties have been correlated to these effects (de Stefano et al. 2012; Mohammadinejad et al. 2019). For example, surface charge of 1.5 nm AuNP was demonstrated to be responsible for the mechanism of cell death in HaCaT cells *in vitro*. Charged AuNP led to disruptions in mitochondrial membrane potential and intracellular calcium levels causing apoptosis, whereas neutral AuNP were associated with necrotic cell death (Schaeublin et al. 2011). Preferential HaCaT apoptosis or necrosis has also been associated with AgNP surface coating and TiO_2_ NP crystal phase (Braydich-Stolle et al. 2009; Bastos et al. 2016). Collectively, these findings assert that surface chemistry/reactivity of metal nanomaterials may be a critical property in determining whether dermal exposure results in irritation responses or sensitization.

The last validated alternative approach to evaluate skin sensitizing potential involves assessment of the potential for an agent to induce upregulation of activation markers (CD 86 and CD 54) on human APC. However, since the recommended cell lines for these assays (THP-1 and U937) are representative of general DC and not skin-specific DC, these studies will be discussed in the *in vitro* section of this review, as they may also apply to respiratory sensitization and augmentation of allergy.

Very few studies have been conducted to investigate metal nanomaterial effects specific to LC. However, topical exposure to < 100 nm AgNP in guinea pigs was shown to increase the number of LC at the site of exposure in a dose- and time-dependent manner (Korani et al. 2011). This observation is relevant to skin sensitization since the concentration of LC present in the skin has been correlated with increased susceptibility to ACD development. Other *in vivo* studies confirmed metal nanomaterials including QD are taken up by LC and subsequently transported to lymph nodes (Jatana et al. 2017b). *In vitro*, associations with LC have been shown to be influ-enced by SiNP size and surface functionalization (Vogt et al. 2006; Rancan et al. 2012). Smaller SiNP size has also been correlated to increased uptake, ROS production, and cytotoxicity to LC *in vitro* (Nabeshi et al. 2010; Yoshida et al. 2014).

A specific observation regarding DC that has implications for ACD and dermal sensitization is that some metal nanomaterials can promote DC cross-presentation. Cross-presentation describes uptake of exogenous antigens and their subsequent processing by pathways normally associated with endogenous antigens. As a result, the exogenous antigen is presented by MHC I molecules to CD8^+^ T-cells, generating the cytotoxic effector cells characteristic of ACD.

Aluminum nanoparticles (AlNP), AuNP, FeNP, and SiNP have all been shown to modify DC antigen cross-presentation capacity (Blank et al. 2011; Li et al. 2011; Hirai et al. 2012; Jiménez-Periáñez et al. 2013; Kang S et al. 2017; Mou et al. 2017; Dong et al. 2018). The mecha-nism of antigen uptake by DC is known to influence the preferential association of antigens with MHC I or II molecules. Small lipophilic haptens associated with skin sensitization often enter APC via passive diffusion and bind cytoplasmic proteins, favoring their processing by endogenous pathways and presentation by MHC I molecules (Rustemeyer et al. 2006). Accordingly, passive diffusion through cell membranes similar to that demonstrated by charged 15 nm AuNP may result in promotion of cross-presentation (Arvizo et al. 2010; Lin et al. 2010; Taylor et al. 2010). Contrarily, receptor-mediated endocytosis of larger antigens has been associated with cross-presentation when uptake occurs by F_c_ and mannose receptors (Blum et al. 2013). In this regard, the adsorption of macromolecules, including immunoglobulins, to the surface of nanomaterials and physicochemical properties associated with the adsorption of proteins may be critically influential in determining the route of antigen processing.

Another major determinant of antigen association with MHC I or II is persistence inside DC. Antigens resistant to degradation in endosomes are more likely to be processed by MHC I pathways (Lin et al. 2008; Humeniuk et al. 2017). Likewise, metal nanomaterials with physicochemical properties capable of compromising lysosomal acidification (dissolution rate, surface reactivity) may promote cross-presentation (Accapezzato et al. 2005; Savina et al. 2006). Similarly, endosomal escape following uptake by DC can result in binding to cytosolic proteins and subsequent perception as an endogenous antigen (Lin et al. 2008). One major mechanism of endosomal antigen release leading to cross-presentation is oxidative stress and lipid peroxidation, causing antigen leakage from compromised endosome membranes (Shen H et al. 2006; Shahbazi et al. 2014; Dingjan et al. 2016). Oxidative stress induced by CuNP, FeNP, and TiO_2_ NP have been shown to cause lipid peroxidation, and these metal nanomaterials have also been associated with enhancing DC cross-presentation (Shukla et al. 2011; Napierska et al. 2012; Manke et al. 2013). Metal nanomaterials have also been associated with the induction of autophagy and production of exosomes by DC, both of which have also been associated with antigen cross-presentation (Moron et al. 2004; Chaput et al. 2006; Crotzer and Blum 2009; Li et al. 2011; Shen T et al. 2018).

### Augmentation of existing or developing skin allergy

Since dermal exposure to metal nanomaterials nearly always occurs simultaneously to other exposures, their potential to augment skin allergy has been investigated using various allergy models. Metal nanomaterial effects on skin allergy have been studied with respect to both T-cell-mediated ACD and IgE-mediated atopic dermatitis. The effects of metal nanomaterials in ACD models have demonstrated findings suggestive of potential effects during both allergic sensitization and elicitation. In one study, subcutaneous exposure to TiO_2_NP 1 hr prior to skin sensitization with dinitrochlorobenzene (DNCB) increased susceptibility of mice to sensitization, as evidenced by a lower concentration of DNCB required to induce sensitization (Hussain et al. 2012; Smulders et al. 2015). The authors noted that although DNCB is known to induce a T_H_1-dominant response characteristic of ACD, exposure to TiO_2_NP resulted in a T_H_2-dominant response in the regional lymph nodes. In a similar study, TiO_2_NP were applied topically 1 day prior to sensitization with DNCB, and the same effect on sensitization as observed (Smulders et al. 2015). A diminished T_H_1 response was observed and TiO_2_NP were detectable in the lymph nodes. Contrarily, SiO_2_NP and AgNP did not induce alterations to DNCB sensitizer potency in the same model.

In another study by (Jatana 2017a), a panel of metal nanomaterials with various physicochemical properties was analyzed for effects on chemical-induced ACD both during sensitization and challenge. When mice were sensitized to dinitrofluorobenzene (DNFB), co-administration of QD did not impact the severity of the challenge response to DNFB, irrespective of particle charge. However, QD administration simultaneous to DNFB challenge did impact the allergic response. Moreover, the effect was dependent on the charge of the materials. The negatively-charged particles suppressed inflammation, whereas the positively-charged materials enhanced ear swelling. The authors confirmed that sensitization to QD did not occur and suggested that variations in skin penetrating capacity of the differently-charged materials was responsible for the observed effects. The conclusions regarding a critical role for nanomaterial size and charge on modulation of ACD elicitation responses is supported by other findings, as well. Suppressive effects on allergic elicitation have also been demonstrated following applica-tion of 20 nm SiNP and <50 nm AgNP-containing cream on ACD reactions to DNFB and 2-deoxyurushiol (Jatana 2017a). Contrarily, exposure to positively-charged functionalized 56 nm SiNP did not augment the severity of oxazolone-induced elicitation responses when topically applied for five consecutive days (Ostrowski et al. 2014).

As highlighted by (Jatana 2017a), ACD responses may be subject to modulation as a result of chemical modifications induced by interactions with metal nanomaterials. In their study, the topical application of nanomaterials was subject to removal prior to application of DNFB. As a result, the particle-specific modulation of allergic skin inflammation was not reflective of blocked adduct formation. Although metal nanomaterials exhibit characteristically increased surface reactivity and catalytic potential, their capacity to impact the chemical properties of skin sensitizing chemicals has not been extensively studied. However, a few studies have demonstrated the potential for such effects to impact both ACD sensitization and elicitation. AlNP and AuNP have been shown to act as non-protein carriers of haptens capable of facilitating the generation of hapten-specific adaptive immune responses *in vivo* (Ishii et al. 2008; Maquieira et al. 2012). Similarly, topical application of ointment containing calcium-based nanoparticles has been shown to capture nickel ions by cation exchange, compromising bioavailability and subsequently preventing the elicitation of nickel-specific ACD (Vemula et al. 2011).

In addition to ACD, metal nanomaterial effects on IgE-mediated atopic dermatitis have also been examined. Atopic dermatitis is generally associated with protein allergens, which under normal circumstances are not capable of penetrating the skin (Smith Pease et al. 2002). However, 100 nm ZnO NP and 5 nm AuNP have been shown to enhance skin penetration by albumin and protein drugs (Huang et al. 2010; Shokri and Javar 2015). Likewise, increased permeability of the skin associated with some metal nanomaterials may represent a mechanism by which exposure may increase susceptibility to atopic dermatitis onset.

Simultaneous exposure to TiO_2_ NP, AgNP, and SiO_2_ NP during sensitization to house dust mite (HDM) in atopic dermatitis models has been associated with an amplification of T_H_2 responses. This effect was shown to be more pronounced with decreasing size with respect to AgNP and SiO_2_ NP, but not for TiO_2_ NP (Yanagisawa et al. 2009; Hirai et al. 2012b; Hirai et al. 2015). Exposure to 5 nm AgNP during sensitization was associated with an augmentation of mast cell activity that resulted in more severe skin lesions that appeared earlier than those induced by 100 nm AgNP (Kang H et al. 2017). Decreases in SiO_2_NP size were also associated with enhanced T_H_2 responses, as evidenced by increased thymic stromal lymphopoeitin (TSLP) and IL-18 production (Hirai et al. 2012b). Decreased particle size has been associated with increased aggravation of atopic dermatitis skin inflammation by nonmetal nanoparticles, as well (Yanagisawa et al. 2010).

Metal nanomaterial-induced modulation of allergic inflammation in the challenge phase of atopic dermatitis has also been demonstrated. Topical application of both 240 and 20 nm ZnO NP resulted in diminished localized inflammation. However, the smaller particle was associated with more pronounced suppression of local inflammation, but simultaneous increases in systemic production of IgE (Ilves et al. 2014).

An interesting observation by Hirai et al. (2015) highlights a potentially critical variation between studies that may explain discordant immune effects induced by similar nanomaterials. distinction between allergy model studies that may contribute to discordant findings. The authors demonstrated that, in their study, exacerbation of allergic sensitization was dependent on co-administration of HDM antigen and SiO_2_ NP. When SiO_2_ NP agglomerates were administered at a site distal to that of the allergen, the modulation of antibody production was no longer observed. The dependence of physical associations between nanomaterial and antigen on the subsequent adaptive immune response has been similarly demonstrated by FeNP. In multiple studies, intravenous administration of 58 nm FeNP 1 h prior to subcutaneous ovalbumin (OVA) sensitization resulted in decreased levels of IgG_1_ and IgG_2_ and suppression of T_H_1 and T_H_17 responses in mice (Shen et al. 2011, 2012; Hsiao et al. 2018). Contrarily, when FeNP and OVA were co-administered intravenously or subcutaneously, enhanced antibody responses were seen in mice (Powles et al. 2017; Shen I et al. 2018). Similar immune-stimulating effects of FeNP have been demonstrated when used as an adjuvant by various exposure routes during immunization to various other antigens in the context of vaccine studies (Pusic et al. 2013; Hoang et al. 2015; Sungsuwan et al. 2015; Neto et al. 2018; Zhao et al. 2018).

The capacity for the same metal nanomaterial to induce divergent immune effects depending on its physical association with antigen likely reflect the different mechanisms of immune modulation associated with prototypical vaccine adjuvants. Some adjuvants exert effects based on “immune modulating” mechanisms. These adjuvants often bear structural resemblance to PAMP, which emphasizes their similar mechanisms of innate immune activation that promote antigen-specific adaptive responses. Since these adjuvants alter the systemic immune environment, their effects are not dependent on physical association with the antigen (Singh and O’Hagan 2003). Contrarily, physical association with antigen is required for adjuvants whose efficacy reflects their capacity to modulate antigen delivery to the immune system. The strength and nature of the adaptive immune response are heavily dependent on the dose and duration of antigen exposure to lymphocytes, so many adjuvants enhance adaptive immune responses by augmenting this process (Johansen et al. 2008).

In accordance with these two divergent mechanisms of sensitization adjuvancy, different physicochemical properties of metal nanomaterials may be implicated in immune effects resulting from each mode of action. For example, simultaneous administration of adjuvant and antigen is often implemented with the intent of depot formation, which increases local antigen retention (Glenny et al. 1931; Demento et al. 2012). Accordingly, metal nanomaterial adjuvant effects dependent on this mechanism are subject to influence by properties involved in antigen trapping, such as size, degree of agglomeration, surface area, and surface charge (Henriksen-Lacey et al. 2010a; Henriksen-Lacey et al. 2010b; Kaur et al. 2012a, 2012b).

### Knowledge gaps in metal nanomaterial effects on skin allergy

Knowledge regarding metal nanomaterial effects on skin sensitization is largely limited to TiO_2_ NP, SiO_2_ NP, and ZnO NP. Though the selective investigation of these metals is likely reflective of their significance to consumer skin exposures, titanium, silver, and zinc are not historically associated with clinically-significant rates of ACD in the general population. Accordingly, the observation that some of these metals may have increased potential to induce skin sensitization in nanoparticulate form raises additional concerns over the lack of investigations into nanomaterials comprised of metals commonly associated with ACD (nickel, gold, cobalt).

## Metal-based nanomaterials and asthma

Respiratory exposure to nanomaterials from naturally-occurring and anthropogenic sources has been taking place for centuries; however, the emergence of engineered nanomaterials and their widespread incorporation into consumer goods pose a risk for inhalation exposures to higher doses of materials with diverse chemical compositions and unique properties (Buzea et al. 2007). In this regard, metal nanomaterials of concern for consumers include many of the materials mentioned above, such as ZnO NP, AgNP, TiO_2_ NP, and SiO_2_ NP, as a result of their incorporation into construction materials, sunscreen sprays, disinfectants, and cosmetic powders, which upon use can lead to their inhalation. Workers are at risk for inhalation exposure to these and other highly-produced metal-based nanomaterials (Table 1) (Nanomaterials Future Markets 2015). Effects of metal nanomaterials on pulmonary immunity and asthmatic conditions have been extensively studied and summarized in this section. In addition to studies reporting adverse immune effects in workers subject to metal nanomaterial inhalation, animal studies that have generated evidence of potential for respiratory sensitization and augmentation of asthmatic conditions are discussed. Likewise, Table 5 summarizes studies characterizing individual metal nanomaterial effects on pulmonary immunity. Table 6 summarizes studies to examine the effects of metal nanomaterial physicochemical properties on asthma. Table 7 highlights some processes involved in respiratory sensitization and elicitation demonstrated to be subject to modulation by metal nanomaterials.

### Human studies demonstrating pulmonary immune effects of metal nanomaterials

A 2014 case study best illustrates the concerns associated with the unknown allergic effects of metal nanomaterials. In the report, a chemist who accidentally inhaled NiNP in the work-place subsequently developed clinical symptoms indicative of IgE-mediated respiratory allergy including throat irritation, nasal congestion, facial flushing, and respiratory distress upon future encounters with NiNP. The chemist also developed previously-nonexistent symptoms indicative of T-cell-mediated ACD in response to non-nanoparticulate forms of nickel in her earrings and belt buckles (Journeay and Goldman 2014). In addition to reinforcing existing concerns over increased potential for allergic sensitization as a result of decreased size, the case also emphasized additional concerns reflective of the unique mechanisms of metal allergy. The case showed that sensitization via one exposure route may not limit future elicitation reactions to the same tissue; moreover, sensitization by metal ions, irrespective of original parent material size, may result in elicitation reactions following exposure to both nano- and bulk-sized metal materials.

Adverse immune effects with implications for allergy have been investigated in human subjects with risk of inhalation exposure to metal nanomaterials in their workplaces. In one study, it was shown that workers of nanomaterial-handling facilities in Taiwan exhibited increased prevalence of sneezing, dry cough, and productive cough compared to workers with no nanomaterial exposures (Liao et al. 2014). Although the workers were employed by facilities handling SiO_2_ NP, Fe_2_O_3_ NP, AuNP, AgNP, and TiO_2_NP, it is unclear whether the observed respiratory effects were mediated by adaptive immune responses specific to the metals, or nonspecific irritant mechanisms. Interestingly, increased rates of ACD were also observed in the workers of the nanomaterial-handling facilities, but the inciting agents were not determined. As such, it is unknown if exposure to the nanomaterials induced sensitization or caused increased susceptibility to ACD development in workers.

Similar studies have also demonstrated that exposure to nanomaterials in the workplace can cause elevations in various immune-related biomarkers indicative of potential effects on allergy (Andujar et al. 2014; Glass et al. 2017; Kurjane et al. 2017) For example, elevations in breath condensate leukotriene (LT) levels were observed in subjects exposed to TiO_2_ NP aerosol (< 100 nm) in their workplaces (Daniela et al. 2016). The exposed workers had elevated levels of LTB_4_, a lipid mediator associated with the recruitment, activation, and prolongation of survival of leukocytes in the lung, as well as multiple cysteinyl leukotrienes (i.e. LTC_4_, LTE_4_, LTD_4_), that are potent mediators of bronchoconstriction (Luster and Tager 2004; Zakharov et al. 2016). Although no alterations in lung function were observed in the exposed workers, elevations in levels of lipid mediators involved in the pathogenesis of asthma suggest the potential for exposure to TiO_2_NP in the workplace to influence the severity of asthmatic conditions.

Collectively, these reports emphasize the potential occupational hazards associated with metal nanomaterials. Although numerous immune markers have been shown to be modulated in workers following inhalation exposure to metal nanomaterials, specific implications for asthma remain unclear. Limitations of human studies arise from inconsistencies between exposure conditions, subject histories, and the requirement for noninvasive, measurable endpoints. Accordingly, the effects of metal nanomaterials on pulmonary immunity and underlying mechanisms have been assessed in animal models wherein controlled dosing, consistent environments, and additional endpoints have helped identify some of the potential underlying mechanisms of metal nanomaterial-induced pulmonary immune effects.

### Evidence for increased potential for respiratory sensitization from animal studies

Assessment of respiratory sensitization presents numerous challenges underscored by the absence of validated *in vivo*, *in vitro*, or *in silico* approaches for identification of potential sensitizers. However, biomarkers with proposed utility for *in vivo* identification of potential respiratory sensitizers following pulmonary exposure include IgE and T_H_2 cytokines (de Jong et al. 2009; Chary et al. 2018). These markers have not been employed for direct evaluation of respiratory sensitization potential by metal nanomaterials; however, numerous studies have reported increased IgE levels following *in vivo* pulmonary exposure to TiO_2_ NP, PtNP, FeNP, AgNP, and ZnO NP (Park et al. 2009; Park et al. 2010; Cho et al. 2011; Huang et al. 2015; Seiffert et al. 2015). Many of the same nanomaterials have also been associated with increased T_H_2 cytokine levels (i.e. IL-4, IL-5, IL-13) following pulmonary exposure (Pettibone et al. 2008; Park 2010; Marzaioli et al. 2014). Although these findings are suggestive of the potential for metal nanomaterials to induce asthma, since the specificity of IgE molecules was not determined in any studies, the capacity for respiratory sensitization remains speculative.

Assessment of respiratory sensitization potential is further complicated by the absence of an AOP specific to the events associated with asthma development. Moreover, discrepancies in some key events involved in asthma inception by LMW and HMW agents indicate the potential requirement for multiple respiratory sensitization AOP. However, many steps are known to be conserved with respect to sensitization of the skin and lungs; knowledge of metal nanomaterial effects on these processes can provide potential insight regarding their potential to cause asthma.

The induction of respiratory sensitization is ultimately dependent on antigen bioavailability. Although it remains unclear whether nano-scale dimensions of metals increase the likelihood for absorption following dermal exposures, the respiratory tract presents a portal of entry known to be increasingly susceptible to smaller materials (Mercer et al. 2018). Nano-materials exhibit a characteristically increased level of “dustiness,” a property which describes the propensity for a material to become airborne following disruption (Evans et al. 2013). Accordingly, the potential for aerosolization and inhalation of metal nanoparticles increases with decreasing size, thereby overcoming one of the limiting steps of respiratory sensitization associated with larger-sized metal particles.

Sensitization of the lungs also requires interactions between the sensitizing agent and APC. The respiratory tract is equipped with an expansive repertoire of defense mechanisms that prevent such interactions, but metal nanomaterials have been shown to have increased capacity to circumvent many of these mechanisms, increasing their potential for uptake by DC. In the upper airways, a layer of mucus lining the lumen functions to trap inhaled antigens and facilitate their translocation out of the trachea by the mucociliary escalator (Moldoveanu et al. 2009). Evasion of the ~5 mm thick mucus layer has been associated with nanomaterial physicochemical properties including size, surface modification, and surface charge (Samet and Cheng 1994; Yang et al. 2008; Liu et al. 2015; Murgia et al. 2016). Generally, hydrophilic, neutrally-charged nanomaterials with smaller diameters have been shown to penetrate mucus to a greater degree than counterparts with opposing properties (Schuster et al. 2013).

In the lower airways, a similar mechanism of antigen neutralization is facilitated by pulmonary surfactant (Chroneos et al. 2010). In addition to optimizing the mechanics of respiration, surfactant contains proteins capable of binding aeroallergens, accelerating their clearance, and preventing their uptake by APC, thereby inhibiting antigen-specific responses (Malhotra et al. 1993; Wang et al. 1996; Hohlfeld 2002; Ruge et al. 2011). Two of these proteins, surfactant protein (SP)-A and SP-D, have been shown to bind to various metal nanomaterials leading to accelerated clearance by phagocytic mechanisms (Ruge et al. 2011). Accordingly, nanomaterials with properties that deter binding to surfactant proteins, such as surface charge, may exhibit increased potential for evasion of clearance by this mechanism, increasing potential for interaction with lung DC (Schulze et al. 2011).

Increased potential for antigen/nanomaterial interaction with DC can also result from evasion of pulmonary macrophage-mediated clearance. Uptake and sequestration of antigen by pulmonary macrophages results in intracellular chemical degradation or physical translocation out of the lungs, preventing their interception by DC (Elder and Oberdörster 2006). Nano-materials can evade clearance by this mechanism by numerous effects. First, since macrophages have been shown to selectively phagocytose nanomaterials according to size, charge, and surface modification, physicochemical properties may contribute to their persistence in the respiratory tract. Their clearance may also be compromised as a result of selective cytotoxic effects on pulmonary macrophages and subsequently fewer viable macrophages capable of neutralizing the nanoparticles. Pulmonary macrophage cytotoxicity has been associated with physicochemical properties including morphology, surface charge, and rate of dissolution (Oh et al. 2010; Hamilton et al. 2014; Shim et al. 2017). Metal nanomaterial-induced alterations in phagocytic activity of pulmonary macrophages, as demonstrated by TiO_2_ NP, ZnO NP, and AlNP, may also contribute to evasion of clearance (Wagner et al. 2007; Liu et al. 2010; Liu H et al. 2013). In addition to compromising the clearance capacity of the phagocytic system on a cellular level, nanomaterials are also associated with maximizing the clearance capacity of the collective phagocytic system. Volumetric loading of alveolar macrophages following inhalation of nanomaterials decreases the efficiency of clearance, extending biopersistence, and increasing the potential for interception by DC (Oberdorster et al. 1992; Blank et al. 2017).

Metal nanomaterial cytotoxic effects on pulmonary macrophages may also promote sensitization by additional mechanisms. Since alveolar macrophages are known to antagonize T_H_2 responses in the lung and downregulate APC functions, cytotoxic effects may disrupt the maintenance of an immunological tolerant state (Tang et al. 2001). Moreover, their depletion leads to significantly increased recruitment of DC and DC precursors to the lungs (Holt et al. 1993; Jakubzick et al. 2006). Numerous metal nanomaterials are also known to trigger the release of alarmins including IL-1β and −1α by alveolar macrophages; in turn, these can activate DC and facilitate sensitization (Braydich-Stolle et al. 2010; Scherbart et al. 2011; Sandberg et al. 2012; Hamilton et al. 2014; Rabolli et al. 2014; Arai et al. 2015).

Similar to their roles in the development of skin allergy, epithelial cells of the respiratory tract are integral in the development of asthma, and their disruption by inhaled materials can have profound influence on the early events of sensitization (Bergamaschi et al. 2006). A major function of airway epithelial cells is to serve as a physical barrier between inhaled agents that deposit in the airway lumen and DC in the epithelium (Hammad and Lambrecht 2015). The importance of barrier integrity in preventing the development of asthma is illustrated by the barrier-disrupting proteolytic activity shared by many aeroallergens with high rates of sensitivity in the population (Kauffman et al. 2006; Lambrecht and Hammad 2012). The frequent observation that metal nanomaterials are capable of inducing cytotoxicity to pulmonary epithelial cells suggests their potential to increase permeability and passage of antigens from the airway lumen to compartments associated with DC.

Airway epithelial cell cytotoxicity has also been associated with the release of alarmins that have potential to promote DC activation and sensitization. Similar to keratinocytes in the skin, the mechanism of cell death can critically influence the nature of the resultant immune response. For example, the necrotic cell death following pulmonary exposure to beryllium results in the release of mediators including cellular DNA, which is recognized as a DAMP by TLR-9, and promotes the unique T_H_1-mediated effects associated with chronic beryllium disease (McKee et al. 2015). NiNP, AgNP, and CoNP have been shown to induce similar necrotic cell death of bronchial and alveolar epithelial cells (Holt et al. 1993; von Garnier et al. 2005; de Haar et al. 2008; Capasso et al. 2014; Ortega et al. 2014). Contrarily, ZnO NP, CuO NP, TiO_2_ NP, and CrNP have all been associated with induction of apoptotic cell death in pulmonary epithelial cells (Park et al. 2007, 2008; Ahamed et al. 2011; Sun et al. 2012; Senapati et al. 2015). This effect may further influence the development of respiratory allergy since uptake of these cells is a property exclusive to CD103^+^ DC, a subset of DC associated with cross-presentation and the subsequent induction of CD8^+^effector responses (Desch et al. 2011).

### Incorporation of metal nanomaterials into asthma models

The impact of metal nano-material exposure prior to sensitization has only been addressed by a few studies using asthma models. Aspiration exposure to ZnO NP, TiO_2_ NP, NiO NP, CuO NP, or SiO_2_ NP 1 d before inhalation sensitization to OVA was followed by inhalation challenge and subsequent assessment of asthmatic severity. Soluble metal nanomaterials (NiO NP, ZnO NP, and CuO NP) were associated with elevations in OVA-specific IgE, whereas insoluble SiO_2_ NP and TiO_2_ NP were not. Subsequent investigations confirmed the importance of metal ion release in the adjuvant effects on sensitization. The increase in OVA-specific IgE production associated with soluble NiO NP was not conserved in response to insoluble NiO microparticles in the same model (Horie et al. 2015). However, ZnCl_2_ also did not exert the same increase in OVA-specific IgE caused by ZnO NP. As a result, it was concluded that continuous ion release from nanoparticles was required for the induction of the observed effects (Horie et al. 2016). Exposure to residual oil fly ash particles prior to allergen sensitization has also been associated with adjuvant effects attributable to soluble metal constituents (Lambert et al. 2000).

Concurrent exposure to metal nanomaterials during allergen sensitization has been explored extensively in order to evaluate the potential adjuvant effects of metal nanomaterials on asthma development. This concept has been explored with respect to both systemic and respiratory sensitization routes, as well as independent and dependent of allergen challenge. In the absence of allergen challenge, evaluation of sensitization achieved by intraperitoneal injection is limited to assessment by systemic markers, such as antigen-specific IgE and cytokine levels. Accordingly, co-administration of AgNP and ZnO NP with antigen has been associated with elevated levels of allergen-specific IgE, as well as increased levels of T_H_2 cytokines (Matsumura et al. 2010; Xu et al. 2013). As demonstrated with SiO_2_ NP, enhanced antibody production has been associated with both increasing dose and decreasing particle size (Toda and Yoshino 2016).

Though few studies have correlated metal nanomaterial properties to adjuvant effects on intraperitoneal sensitization independent of allergen challenge, existing findings are conducive with studies using larger metal particles and nonmetal nanoparticles (Naim et al. 1997; Granum 2001b). The impact of the most extensive number of physicochemical properties with respect to adjuvant effects on OVA sensitization use polystyrene nanoparticles (PSP). Nygaard et al. (2004) used PSP ranging from 58 nm to 11.4 μm to evaluate the influence of particle size, mass, surface area, and particle number. Similarly, Granum et al. (2000) used six sizes of spherical PSP to administer doses with constant mass (12.25 μg), size (0.1 μm), particle number (8 × 10^10^), or surface area (1300 cm^2^). Both studies showed that serum OVA-specific IgE levels best correlated with particle number and surface area (Granum et al. 2001a; Nygaard et al. 2004).

Similar adjuvant effects have been observed following respiratory sensitization and co-exposure to TiO_2_ NP, SiO_2_ NP, and ZnO NP (de Haar et al. 2006; Huang et al. 2015). Increases in OVA-specific IgE and T_H_2 cytokine levels were similarly associated with decreasing size of SiO_2_ NP (Yoshida et al. 2011). Moreover, SiO_2_NP surface properties were shown to impact sensitization independent of allergen challenge. Intranasal exposure to three variations of SiO_2_ NP (spherical, mesoporous, and PEGylated) simultaneous to OVA sensitization exacerbated pathological changes, inflammatory cell influx, and T_H_2 cytokine responses. These effects were specific to the unique surface chemistry of each type of SiO_2_ NP, but the most severe responses were associated with the nanoparticle with the highest surface area (Han et al. 2016).

The absence of allergen challenge in these studies helps elucidate the direct effects of metal nanomaterials on sensitization processes. However, another approach to evaluate the same effect involves evaluation of allergic parameters collected in response to allergen challenge. Studies utilizing this approach have similarly demonstrated enhanced asthmatic responses in OVA-challenged mice when intraperitoneal sensitization occurred simultaneous to TiO_2_ NP and ZnO NP (Larsen et al. 2010; Roy et al. 2014a; Roy et al. 2014b; Mishra et al. 2016).

Although the observed effects may reflect residual impacts of metal nanomaterial respiratory exposure during sensitization, similar adjuvant effects on elicitation responses have been observed following respiratory sensitization and simultaneous metal nanomaterial exposure. Simultaneous administration of SiO_2_NP, CeO_2_NP, QD, and TiO_2_NP with allergen during sensitization led to enhanced asthmatic response severity, as measured by antigen-specific antibody levels, inflammatory cell influx, and T_H_2 cytokine levels after challenge (Brandenberger et al. 2013; Meldrum et al. 2018; Vandebriel et al. 2018; Scoville et al. 2019). Studies using similar sensitization procedures and endpoints have also implicated TiO_2_ NP crystal structure in adjuvant effects on sensitization (de Haar et al. 2006; Vandebriel et al. 2018).

Metal nanomaterial exposure has also been incorporated into the challenge phase of asthma to evaluate potential modulation of asthmatic responses in established asthmatic conditions. Although some metals, including CuO NP, have been exclusively shown to induce significant aggravating effects on elicitation responses, others, including AuNP, appear to exert protective effects on asthmatic responses (Barreto et al. 2015; Park et al. 2016; Omlor et al. 2017). Contrarily, other metal nanomaterials, including TiO_2_ NP, have been associated with divergent effects on allergen challenge that appear increasingly susceptible to variation during this phase of asthma. Effects have been reported to be differentially induced according to dose, duration of exposure, and endpoints of assessment (Rossi et al. 2010; Hussain et al. 2011; Jonasson et al. 2013; Kim et al. 2017).

Similarly, after OVA sensitization via intraperitoneal injection, AgNP exposure during allergen challenge has been reported to induce various aggravating and attenuating effects on allergic inflammation. Inhalation exposure to 6 nm AgNP was shown in multiple studies to suppress inflammatory cell influx, airway hyper-reactivity (AHR), mucus hypersecretion, and other measures of asthmatic responses (Park, Kim, Jang, et al. 2010; Jang et al. 2012). Contrarily, in another study with very similar exposure conditions, 33 nm AgNP caused increased airway response, inflammatory cell influx, and OVA-IgE levels over control animals (Chuang et al. 2013; Su et al. 2013). The discrepancies between these studies may be attributable to AgNP size difference, as well as potential variations in particle coating, both of which have been associated with differ-ential effects on asthmatic responses (Alessandrini et al. 2017). Additionally, the first two studies used the T_H_1-dominant C57BL/6 mouse strain, whereas the second used a T_H_2-biased BALB/c strain (Jones et al. 2013). Strain-specific immune responses following respiratory exposure to metal nanomaterials during allergen challenge have been demonstrated in other studies, as well (Gustafsson et al. 2014).

Studies using SiNP and ZrO NP with variations in surface properties demonstrate that when administered during allergen challenge, surface properties of nanomaterials can differen-tially aggravate allergic inflammation (Marzaioli et al. 2014; Park, Sohn, et al. 2015; Vennemann et al. 2017). It has been suggested that particles with higher oxidant potential amplify asthmatic inflammation to a greater degree, which would implicate physicochemical properties such as surface modification in these effects (Li et al. 2009).

### Potential mechanisms of asthma augmentation by metal nanomaterials

Although asthma models have characterized the potential effects of metal nanomaterial exposure on asthmatic processes, many of the underlying mechanisms of these observed effects remain unclear. However, findings from other studies suggest several mechanisms may be associated with the observed effects of metal nanomaterials on the augmentation of asthma.

Respiratory exposure to metal nanomaterials may increase susceptibility to sensitization by aeroallergens by similar mechanisms previously proposed to contribute to their respiratory sensitization potential. Release of alarmins by airway epithelial cells and resident immune cells, disruption of the T_H_1/T_H_2 balance in the lung, and amplification of oxidative stress by metal nanomaterials may also generate adjuvant effects on sensitization. Similarly, FeNP, TiO_2_ NP, and SiNP have all been shown induce the release of T_H_2 cytokines including IL-33, TSLP, GM-CSF, and IL-25 by airway epithelial cells, which are known to promote DC maturation (Hussain et al. 2009; Val et al. 2009; Mano et al. 2013; Park, Sohn, et al. 2015).

Evidence also suggests metal nanomaterial exposure can modulate inflammatory pheno-types of existing asthmatic conditions. Two major heterogeneous asthma phenotypes differ based on the presence of neutrophil (T_H_1/T_H_17)- or eosinophil (T_H_2)-dominated inflammation (Fahy 2009; Yu and Chen 2018). Particulate and soluble metals are known to differentially impact the nature of existing allergic airway inflammation by skewing this balance (Schneider et al. 2012). Dissolution kinetics also appear influential in this regard, as CoNP, NiNP, ZnO NP, and CuO NP and their corresponding ions have been shown to differentially recruit eosinophils and neutrophils to the lungs of rats after exposure (Cho et al. 2011; Jeong et al. 2015).

Modulation of elicitation response severity by metal nanomaterials may emerge as a result of modulation of mast cell activity. As a major effector cell in IgE-mediated allergic responses, mast cells have been shown to be potential targets of metal nanomaterial-induced adverse effects *in vitro* (Feltis et al. 2015; Johnson et al. 2017; Alsaleh and Brown 2018). AgNP, CuO NP, SiO_2_ NP, and TiO_2_ NP have all been shown to induce IgE-independent mast cell degranulation depending on physicochemical properties including size, surface area, charge, shape, and the presence of adsorbed surface proteins (Marquis et al. 2011; Aldossari et al. 2015; Alsaleh et al. 2016; Johnson et al. 2017). Modulation of IgE-dependent mast cell degranulation has also been demonstrated by some of the same nanomaterials. TiO_2_ NP, AuNP, CeO_2_ NP, ZnO NP, and FeNP have been shown to modulate interactions between allergen and surface-bound IgE molecules, interfering with dimerization and subsequent degranulation (Huang et al. 2009; Ortega et al. 2015). Similarly, mast cell uptake of metal nanomaterials has been associated with modulation of intracellular calcium signaling involved in mast cell degranulation (Amin 2012; Chen et al. 2012). Accordingly, differential ion release by bulk ZnO particles, ZnO NP, and soluble ZnSO_4_ has been correlated with the propensity for OVA-sensitized rat mast cells to degranulate when co-exposed with OVA (Yamaki and Yoshino 2009; Feltis et al. 2015).

Furthermore, TiO_2_ NP and AuNP have been shown to alter the exocytic kinetics of granule secretion by mast cells (Marquis et al. 2009). The qualitative and quantitative profile of granule contents has also been shown to be subject to modulation by some metal nanomaterials. The number of molecules per granule has been shown to be impacted by SiO_2_ NP as a function of porosity and surface area (Maurer-Jones et al. 2010). Variations in vesicle mediator content has been shown to be augmented by AuNP (Marquis et al. 2009). Since the contents of mast cell granules contribute to vascular permeability and inflammatory cell recruitment, these alterations can greatly impact the severity of allergic elicitation (Dudeck et al. 2011; Weber et al. 2015).

Aggravation of existing asthmatic conditions may also involve non-immunological mechanisms, such as metal nanomaterial-induced alterations to normal physiological processes. For example, increased mucus production by epithelial cells is a hallmark symptom of the early and late phase asthmatic response (Erle and Sheppard 2014). The observation that TiO_2_NP and CuONP both increased mucin secretion in human epithelial cells suggests potential to exacerbate asthmatic conditions by contributing to obstruction of airways (Chen et al. 2011; Park et al. 2016). Similarly, TiO_2_ NP, AuNP, and AgNP have been shown interfere with optimal pulmonary surfactant functioning, which can cause AHR and increased resistance to airflow (Hohlfeld et al. 1999; Hohlfeld 2002; Bakshi et al. 2008; Schleh et al. 2009; Zhang et al. 2018). AHR may also be modulated by metal nanomaterials as a result of alteration of airway smooth muscle (ASM) contractility. ZnO NP, CuO NP, and TiO_2_ NP have all been shown to alter human ASM mechanical function *in vitro* (Berntsen et al. 2010). Similarly, CoFe_2_O_4_ NP were shown to potentiate both histaminergic and cholinergic ASM contractility *in vivo*, which has the capacity to exacerbate symptoms of asthma associated with bronchoconstriction (Kapilevich et al. 2012).

Metal nanomaterial exposure may also exacerbate established asthmatic conditions by accelerating the progression of pathological alterations associated with chronic asthmatic conditions. The repetitive induction and resolution of inflammation induced by asthmatic elicitation leads to anatomical alterations referred to as airway remodeling (Fehrenbach et al. 2017). Histological indicators of these alterations have been reported to be exacerbated by various metal nanomaterials including SiNP (Han et al. 2011). Similarly, cellular indicators of accelerated airway remodeling have been implicated in response to many metal nanomaterials. For example, fibroblast accumulation and increased extracellular matrix deposition is a common contributor to airway remodeling and has been observed in response to NiNP, SiO_2_ NP, and CeO_2_ NP exposure (Warner and Knight 2008; Han et al. 2011; Ma et al. 2012; Armand et al. 2013; Glista-Baker et al. 2014).

### Knowledge gaps in metal nanomaterial effects on asthma

Despite the known capacity for many metals to induce IgE-mediated asthma following inhalation, the potential for metal nanomaterials to induce sensitization of the respiratory tract remains completely unknown. Several other interesting aspects of pulmonary immunity have not been widely addressed with respect to metal nanomaterials, and may have relevance to current observations regarding their effects on asthma. The microbiome is known to significantly impact numerous aspects of allergic disorders, and while some metal nanomaterials associated with antimicrobial activity have been shown to alter the pulmonary microbiome, the implications for asthma remain unknown (Alessandrini et al. 2017; Poh et al. 2018). Similarly, the effects of metal nanomaterials on innate lymphoid cells also remain largely unstudied, but should not continue to be neglected, given the importance of this cell type in allergic disorders. Finally, the capacity for metal nanomaterials to disrupt or prevent the development of immunological tolerance has not been explored, and may be influential in both phases of asthmatic conditions.

## Effects of metal nanomaterials on immune cells and allergic processes *in vitro*

*In vivo* studies have demonstrated the capacity for metal nanomaterials to augment numerous immunological processes that result in functional implications for allergic disease. However, *in vitro* investigations have helped elucidate some of the underlying mechanisms responsible for *in vivo* observations. In this section, major findings regarding the role of metal nanomaterial physicochemical properties on molecular and cellular processes with implications for both ACD and asthma are summarized.

### Effects on antigen immunogenicity

Many physicochemical properties associated with the molecular and cellular processes that confer antigen immunogenicity are subject to alteration following interactions with constituents of their environment. In this regard, the immunogenicity of metal nanomaterials may be significantly altered as a result of biocorona formation. Following entry into biological media, macromolecules present in the media interact with and adsorb to the surface of nanomaterials within minutes, forming a layer that defines the bio-identity of the nanomaterial (Corbo et al. 2016). The qualitative profile of adsorbed constituents and quantitative strength of association have been shown to be influenced by nanomaterial properties including size, charge, morphology, surface modification, and hydrophilicity, among other properties (Lundqvist et al. 2008; Dobrovolskaia et al. 2014).

Independent of adsorbed constituents’ identities, macromolecule associations with metal nanomaterial surfaces may induce alterations in physicochemical properties associated with their bioactivity (Dobrovolskai et al. 2009b; Yin et al. 2015). For example, protein adsorption to 25 nm FeNP was associated with a 5-fold increase in hydrodynamic size, which can impact a number of biological effects, such as propensity for cellular uptake (Calatayud et al. 2014). Similarly, adsorption of proteins can mask reactive surfaces of metal nanomaterials, attenuating ROS generation, and subsequently inhibiting a major biochemical mechanism involved in the release of alarmins (Ilinskaya and Dobrovolskaia 2016).

Contrarily, surface adsorption of macromolecules may alter the biological activity of metal nanomaterials in a manner that is dependent on the adsorbed constituent profile. Endo-genous proteins, including immunoglobulins, cytokines, and complement proteins are all constituents of the serum and lung lining fluid known to bind metal nanomaterial surfaces (Neagu et al. 2017). The binding of complement protein C3b and IgG to nanomaterial surfaces has been shown to confer recognition by complement and Fc receptors, accelerating clearance by phagocytes, and limiting the potential of the nanomaterial to induce other immune effects (Beduneau et al. 2009; Moghimi et al. 2012; Frohlich 2015). Adsorption of exogenous agents can also lead to alterations in nanomaterial immunogenicity. One of the most notable examples is endotoxin (LPS), a frequent microbial contaminant of nanomaterial surfaces that is capable of activating innate immune cells, and inducing pro-inflammatory signaling via the TLR-4 pathway. Adsorption of LPS to nanomaterial surfaces has been shown to enhance inflammatory responses to many metal nanomaterials by lung epithelial cells, and various immune cells (Shi et al. 2010; Liu et al. 2012; Bianchi et al. 2017; Li et al. 2017; Ko et al. 2018). The chemical structure of LPS favors its adsorption to hydrophobic, positively-charged metal nanomaterial surfaces, indicating a role for physicochemical properties such as surface modification in the propensity for associations with immunogenic exogenous molecules such as LPS (Gorbet and Sefton 2005; Li et al. 2017).

Although biocorona formation can augment the immunological fate of a nanomaterial, the interactions may also facilitate alterations in immunogenicity of the adsorbed constituents. Deng et al. (2010) showed that functionalized AuNP were capable of binding fibrinogen independent of nanoparticle size, but certain sizes of AuNP induced conformational changes in the protein. Subsequent alterations in protein structure conferred its recognition by the Mac-1 receptor, subsequently activating NF-κB signaling in innate immune cells. Similarly, Bastus et al. (2009) demonstrated that while macrophages did not recognize AuNP or two biomedically-relevant peptides individually, their conjugation facilitated recognition by TLR-4 and the subsequent induction of pro-inflammatory cytokine production. Since these signaling pathways play critical roles in many of the adjuvant effects mentioned in previous sections, interactions between metal nanomaterials and host proteins can generate novel sources of immunogenicity that may promote allergic processes.

The generation of metal antigens *in vitro* has been shown to be impacted by the unique physicochemical properties of metal nanomaterials. The size-specific increase in surface energy of TiO_2_ NP was shown to promote associations between the metal and human serum albumin, resulting in increased bioavailability (Vamanu et al. 2008). This altered propensity for inter-actions with host proteins contributed to the observation that titanium antigens generated from ionic and nanoparticulate forms of TiO_2_ induced differential proliferation of CD4^+^ and CD8^+^ T-lymphocytes *in vitro* (Hol et al. 2018).

### Effects on processes involved in sensitization

As the primary APC involved in allergic sensitization, the capacity for metal nanomaterials to modulate DC activity represents a prominent mechanism by which allergic sensitization can be impacted (Jia et al. 2018). Since many sensitizing agents are known to induce both antigen-specific and nonspecific signals to DC following exposure, DC activation is a step of the dermal sensitization AOP used for *in vitro* identification of potential sensitizers. Accordingly, the h-CLAT method has been validated for use by the OECD to determine the skin sensitizing potential of agents. In this assay, undiffer-entiated THP-1 human monocytic leukemia cells are exposed to an agent for 24 h and their activation status is subsequently assessed by quantification of CD86 and CD54 activation marker expression (Ashikaga et al. 2002, 2006; Nukada et al. 2012).

The h-CLAT assay has not been employed to evaluate the sensitizing potential of any metal nanomaterials; however, several studies have investigated metal nanomaterial effects on undifferentiated THP-1 cells following a 24-h exposure, and reported activation marker expression. Accordingly, up-regulation of CD86 expression was observed following exposure to surface-modified FeNP, SiO_2_ NP, and mixed-metal alloy nanoparticles (Liu, Y et al. 2013). de Marzi et al. (2017) exposed THP-1 cells to a wide range of SiO_2_ particle sizes (10–1430 nm); while all particles promoted activation marker expression, the 240 nm SiO_2_ particles induced the greatest degree of CD80 expression. Similar findings were reported by an investigation that assessed the potential for metal debris released from orthopedic implants to trigger immune activation. Both ~2 mm cobalt-chromium-molybdenum alloy particles and soluble metal ions induced elevations in THP-1 co-stimulatory molecule expression, suggesting that a wide range of metal particle sizes have the capacity to induce immune effects involved in allergic sensitization (Caicedo et al. 2010; de Marzi et al. 2017). Contrarily, no elevations in THP-1 expression of CD86 or CD54 were observed in response to 100 nm AgNP exposure (Galbiati et al. 2018).

Though CD86 and CD54 are the validated biomarkers indicative of sensitizing potential in the THP-1 line, limited reports have evaluated these specific markers following metal nanomaterial exposure. However, other markers indicative of DC activation, such as MHC II, CD11b, CD14, CCR2, and CCR5 have been reported to be up-regulated in response to exposures to ZnO NP and FeNP (Prach et al. 2013; Matuszak et al. 2015). Similarly, modulation in expression of 60 genes – several of which were correlated to monocyte differentiation and maturation – were observed in response to PtNP exposure (Gatto et al. 2018).

The THP-1 cell line has also been used to identify potential skin sensitizers *in vitro* based on a unifying property of rapid ROS production following exposure to skin sensitizing chemicals (Miyazawa and Takashima 2012). A similar response has been demonstrated in the cell line following exposure to < 100 nm silver-copper alloy nanoparticles, AgNP, CoO NP, PdNP, and NiNP (Monprasit et al. 2018). The degree of ROS production by THP-1 cells has been correlated to properties including particle size and corona presence, as well as exposure dose and duration (Foldbjerg et al. 2009; Casals et al. 2011; Neubauer et al. 2015). Subsequent activation of the p38 MAPK signaling pathway, alterations in expression of *HMOX1* and other oxidative stress genes have also been used as *in vitro* biomarkers suggestive of sensitizing potential. Numerous metal nanomaterials have been associated with these effects on THP-1 cells, which suggests their potential to activate DC and promote sensitization (Mohamed et al. 2011; Khatri et al. 2013; McConnachie et al. 2013; Boonrungsiman et al. 2017).

The potential for metal nanomaterials to induce DC activation has been more extensively examined using primary DC than the cell lines used in the validated assays (Kang and Lim 2012). Although the expression of activation markers in murine bone marrow-derived DC (BMDC) or human monocyte-derived DC (MDDC) has not been validated by OECD for use in determining sensitization potential *in vitro*, several studies have reported their capacity to accurately predict sensitizers (Tuschl et al. 2000; Pepin et al. 2007). Accordingly, TiO_2_ NP, ZnO NP, and SiO_2_ NP have been associated with increased expression of CD80 and CD86 by murine BMDC with respect to size, surface chemistry, and crystallinity (Palom€aki et al. 2010; Heng et al. 2011; Winter et al. 2011; Zhu et al. 2014; Winkler et al. 2017; Vandebriel et al. 2018).

Nanomaterial-induced modulated DC activity can also influence sensitization indepen-dent of their capacity to induce phenotypical maturation. For example, uptake of AuNP and AgNP resulted in an enhanced capacity for DC maturation in response to other immune stimuli (Orlowski et al. 2018). This finding suggests uptake of antigens normally incapable of activating DC may trigger their maturation in the presence of nanomaterials. In addition, accumulation of metal nanomaterials has been proposed to interfere with antigen processing and presentation by DC (Thiele et al. 2003; Humeniuk et al. 2017).

Polarization of DC and the subsequent preferential generation of T_H_1/T_H_2 effector T-cells is another step in the development of allergy that has been shown to be susceptible to modulation by nanomaterials. Dermal and respiratory sensitizers are associated with divergent oxidative stress responses that induce selective alterations in three major signaling pathways responsible for DC polarization (Mizuashi et al. 2005; Antonios et al. 2009). Polarization of DC towards T_H_1-promoting activity has been associated with the propensity for skin sensitizers to react with cytoplasmic glutathione following which, rapid depletion leads to ROS accumulation. The rapid induction of oxidative stress induced by contact sensitizers is responsible for the selective activation of the p38 MAPK and JNK signaling pathways within minutes of encounter (Nakahara et al. 2006). Contrarily, polarization of DC towards T_H_2-dominant responses has been associated with delayed induction of oxidative stress resulting from the preferential association of respiratory sensitizers with intracellular amine groups (Ferreira et al. 2018). Subsequently, selective activation of the NF-κB and ERK pathways occurs.

Knowledge of these pathways and their differential activation explain the observation that metal nanomaterials with opposing catalytic properties induce different polarization profiles in DC *in vitro*. The oxidant capacity of TiO_2_ NP resulted in potentiation of DC maturation leading to a T_H_1-biased responses, whereas treatment with the anti-oxidant surface activity of CeO_2_ NP resulted in secretion of anti-inflammatory IL-10 and a T_H_2-dominant T-cell profile (Schanen et al. 2013). FeNP, AuNP, and GdNP have also been associated with modulation of DC polarization *in vitro* with respect to size and surface chemistry (Yang et al. 2010; Vallhov et al. 2012; Tomi c et al. 2014; Hoang et al. 2015).

### Effects on processes involved in elicitation of allergy

Metal nanomaterials have been shown to have potential to influence elicitation reactions specific to both metals and environmental proteins *in vitro*. With respect to metal allergy, PBMC isolated from women with established allergic sensitivity to palladium were challenged with either 5–10 nm PdNP or palladium salts *in vitro* (Reale et al. 2011). Variations in TNFα and IL-10 release were noted between exposures, indicating a potential role for metal solubility on metal-specific allergy elicitation. With respect to environmental allergens, basophils isolated from patients with established sensitivity to common environmental allergens including birch pollen, timothy grass pollen, and house dust mite were exposed to AuNP-conjugated with corresponding allergenic proteins. Stable coronas were formed by all three allergens, but binding of allergen to AuNPs caused enhanced activation of basophils in response to house dust mite challenge, as well as birch pollen in some individuals (Radauer-Preiml et al. 2016).

Although lymphocyte cytotoxicity is an immunotoxic effect most often associated with immunosuppression, as major effector cells of both IgE and T-cell-mediated allergic responses, this effect has potential to impact allergic disorders, as well. Accordingly, many metal nano-materials have been shown to be cytotoxic and genotoxic to human and murine lymphocytes *in vitro*. Interestingly, T- and B-lymphocytes have been shown to be more resistant to adverse effects of ZnO NP compared to other immune cell types (Hanley et al. 2009). Although ion release from ZnO NP and PdNP was correlated to cytotoxicity and alteration in gene expression, DNA damage induced by CoNP was shown to be more severe than that induced by Co ions (Jiang et al. 2012; Tuomela et al. 2013; Petrarca et al. 2014; Simon-Vazquez et al. 2016). Susceptibility to cytotoxicity was shown to be reflective of cell cycle status, explaining the finding that memory T-cells were more sensitive to metal nanomaterial effects compared to naïve T-cells (Hanley et al. 2009; Shahbazi et al. 2013).

Modulation of T-lymphocyte activity by metal nanomaterials also has the potential to significantly influence allergic processes. PdNP, AuNP, CoNP, and GdNP have all been shown to induce differential T_H_1/T_H_2-biased cytokine production by lymphocytes *in vitro* dependent on size, solubility, and hydrophobicity (Petrarca et al. 2006; Liu et al. 2009; Boscolo et al. 2010; Moyano et al. 2012). FeNP suppressed the activity of Kv1.3 channels, which suggests a potential mechanism of lymphocyte cell signaling modulation (Yan et al. 2015). Additionally, delays in proliferation, altered mitogen responses, and morphological changes have also been observed by lymphocytes following exposure to various metal nanomaterials (Shin et al. 2007; Beer et al. 2008; Liptrott et al. 2014; Devanabanda et al. 2016).

Knowledge regarding effects of metal nanomaterials specifically on B-cells *in vitro* is limited to AuNP, which have been shown to be size-dependently taken up by B-cells, causing alterations in NF-κB and blimp1/pax5 signaling pathways, and altered secretion of immune-globulins in a size-dependent manner via (Sharma et al. 2013; Lee et al. 2014). The potential for metal nanomaterials to directly alter B-cell processes, such as antigen-specific interactions with T-cells, isotype switching, and affinity maturation, events critical to their effector functions in IgE-mediated allergic disorder such as asthma, remains largely unstudied (Luo et al. 2015).

### *Knowledge gaps in metal nanomaterial effects on immune cells and processes* in vitro

Formation of the nanomaterial biocorona has been almost exclusively investigated with respect to the adsorption of proteins. However, nanomaterials are also subject to interactions with other macromolecules present in biological fluids, such as nucleic acids and lipids. Adsorption of these molecules may have notable impacts on the immune effects of nanomaterials since different nucleic acids are alarmins recognized by PRR and lipid mediators play critical roles in many aspects of allergic disorders (Schauberger et al. 2016; Muller et al. 2018). Accordingly, more research should be directed towards investigating the biological implications of surface-adsorbed macromolecules other than proteins. Similarly, it has been suggested that metal nanomaterials may act as soluble or particulate antigens, but the dynamics of metal antigen generation remains largely unstudied with respect to nanoparticles.

## Important considerations for future metal-based nanomaterial allergy studies

The potential for metal-based nanomaterials to induce immune effects with implications for allergic disease following exposure by routes other than dermal contact or inhalation is a significant knowledge gap. Nanomaterials are being increasingly incorporated into foods, beverages, supplements, and packaging, rendering ingestion exposures an increasing concern (Chaudhry et al. 2008). Ingestion of metal nanomaterials has been associated with altered B-cell distribution, increased levels of IgE and IgG, and splenic toxicity, but the implications of these effects on allergy are unknown (Park et al. 2010a; Kim et al. 2014; Sheng et al. 2014). Similar immune effects have been observed following systemic administration of various metal nanomaterials. Although most current uses for metal nanomaterials are not likely to result in systemic exposures, some nanomaterials with expanding biomedical applications present a concern. The significance of this knowledge gap is demonstrated by the numerous adverse effects in patients administered Feraheme (ferumoxytol), an intravenously-administered iron replacement product containing 17–31 nm colloidal Fe_3_O_4_NP (Lu et al. 2010). In the 5 years following approval for use by the Food and Drug Administration (FDA) in 2009, 79 anaphylactic reactions were reported, of which 19 were fatal.

As more studies are conducted to advance our understanding of the effects of metal nanomaterials on allergic disease, it should be recognized that accurate assessment is dependent on the evaluation of sample contamination with endotoxin (Dobrovolskaia et al. 2009a). As a result of production and handling in non-sterile conditions, engineered nanomaterials are often carriers of impurities including LPS, a potent immunoadjuvant (Smulders et al. 2012). Accordingly, the presence of endotoxin on surfaces of metal nanomaterials could generate a subsidiary but sufficient amount of immunostimulation required to induce allergic sensitization to the metal itself, or to other allergens. Consequently, exposure to immunologically inert metal nanomaterials contaminated with LPS may lead to misidentification of such agents as sensitizers or adjuvants. Many of the studies published prior to this development do not report the presence or absence of endotoxin in samples, and results should be interpreted with caution.

Another consideration for future studies is that the successful correlation of metal nanomaterial physicochemical properties with mechanisms of toxicity is limited by the accurate assessment and reporting of test material characterization. Although thorough material character-ization has become recognized as an indispensable step in nanotoxicity studies, discrepancies in property terminology, evaluation methods, property reporting, and the biological relevance of measured properties complicate comparisons between studies. A notable example of inconsistent property terminology is the tendency for non-discriminate reporting of aggregates and agglomerates of primary particles. The irreversible bonds of aggregates and reversible bonds of agglomerates can impact the effective dose surface area, degree of primary particle dissociation, and other properties that dictate *in vivo* biological effects (Keene and Tyner 2011; Gualtieri et al. 2012; Sharma et al. 2014). Another property subject to inconsistent reporting is nanomaterial surface area. Differences in the material state and method of assessment can generate results representative of different parameters, including volume-specific, geometric, or specific surface area SSA (van Doren et al. 2011; Wohlleben et al. 2017). Although these metrics are often similarly reported by studies, they have been correlated to notable variations in toxic potential (Sager et al. 2016). The use of multiple assessment methods and disclosure of potential sources of measurement variation between studies will help accurately compare the impact of properties on toxic potential in future studies.

Accurate evaluation of nanomaterial physicochemical properties and their correlation to toxic effects has become increasingly relevant as emerging nanomaterials challenge the efficacy of traditional occupational exposure limits (OEL). The majority of respiratory occupational exposure limits do not discriminate for material size, so as new metal-based nanomaterials emerge, they are subject to the same mass-derived values enforced for other materials of the same elemental composition. This issue is proving problematic as nanotoxicity studies continue to demonstrate that mass may not be the best dose metric for prediction of pulmonary toxicity (Schmid and Stoeger 2016). Moreover, as demonstrated by the studies summarized here, metal nanomaterial properties other than mass have been correlated to immune effects following respiratory exposure. Accordingly, size nonspecific OELs may be ineffective in protecting workers from both nanomaterial-induced pulmonary effects, as well as subsequent immune effects (Schulte et al. 2010). This concern has become increasingly recognized, as NIOSH has recommended size-specific exposure limits for TiO_2_. Despite the recommended time-weighted average exposure limits of 2.4 mg/m^3^ for fine TiO_2_ and 0.3 mg/m^3^ for ultrafine and nanoscale TiO_2_, the agency responsible for regulating compliance with its own established limits(i.e. OSHA) has not yet adopted size-specific OEL values for TiO_2_ (NIOSH 2011).

## Conclusions

Although there is a growing amount of toxicological data demonstrating the vast potential for adverse immune effects following exposure to metal nanomaterials, advancements in understanding their interactions with biological systems have allowed for their unique characteristics to be harnessed for beneficial applications, as well. Numerous studies have demonstrated the potential utility of metal nanomaterials for novel vaccine adjuvants, drug delivery vehicles, diagnostic approaches, and immunotherapies. However, to optimize the use of metal-based nanomaterials for these and other advantageous purposes, a more complete understanding of their systemic immune effects, mechanisms of immunomodulation, and capacity to induce allergic sensitization is needed.

## Figures and Tables

**Table 1.** Metal nanomaterial production rates and corresponding applications.

Nanomaterial	Global production volume (tons)	Applications
Silicon dioxide	185,000–1,400,000	Nanocomposite filler, cement additive, drug delivery, cosmetics
Titanium dioxide	60,000–150,000	Ceramics, sunscreens, construction, energy, cosmetics
Zinc oxide	32,000–36,000	Sunscreen, LED and LCD displays, antimicrobial, water filtration
Aluminum oxide	5000–10,100	Drilling equipment, energetic fuels, and additives, filtration
Cerium oxide	880–1400	Catalysts, slurry polishing, UV absorption, anti-corrosion additive
Copper oxide	290–570	Heat transfer, batteries, antimicrobials, sensors, semiconduction
Silver	135–420	Biomedicine, antimicrobial textiles, cosmetics, conductive inks
Antimony tin oxide	120–225	Electronics, composites, coatings, research
Zirconium oxide	80–300	Heat-resistant coatings, ceramics, catalysts, dental composites
Iron oxide	9–45	Ground and wastewater cleanup, color imaging, drug delivery
Nickel	5–20	Ceramic additive, catalyst, energy absorption, electronics
Cobalt oxide	5–10	Electronics, catalysts, drug delivery, solar energy absorption
Quantum dots	4.5–9	Photovoltaic devices, photodetecting devices, electronics
Manganese oxide	2–3.5	Bleaching agent, biomedical diagnostics, plastics additive
Gold	1–3	Data storage, gene therapy, biosensors, fuel cell additive
Palladium	–	Hydrogen sensor, antimicrobial activity, catalysts

Metal nanomaterials, various applications, and reported global production volume for 2014, reported in tons. Adapted from Metal and Metal Oxide Nanomaterials Future Markets Report, Table 1 (Nanomaterials Future Markets 2015).

**Table 2.** Summary of major findings from studies characterizing the effect of metal nanomaterials on dermal allergy grouped by metal

Metal	Author/year	Material	Size	Animal or cell type	Model	Exposure route	Dose	Findings
Aluminum	Varlamova et al. 2015	AI2O3	–	M/F BALB/c, CBA/CaLac, out-bred mice and guinea pig	LLNA	Subcutaneous, intramuscular, intravenous, intra-dermal injection		No intensification of anaphylaxis systemic reaction, no inflammatory reaction to ConA, no delayed allergic reaction, no redness or edema at site of application
	Brown et al. 2008	AI2O3	50–120 nm	HaCaT human keratinocytes		In vitro	10–10,000 μg/mL	24 h exposure resulted in IL-8 expression, IL-1α release, indicating potential for irritation or sensitization
Cobalt	Choet al. 2012	Co3O4	18.4±5.0nm	F C57BL/6 mouse	OVA	Subcutaneous injection	25 μg	Balanced Th1/Th2 response when used as adjuvant, causing higher specific lgG2c and IgGI and less IgE
Gold	Ishii et al. 2008	Au	5.2±1.3nm	F Japan White Rabbit		Intradermal injection	1 mg	Azobenzene dye hapten conjugation to AuNP led to high yield of IgG specific to the agent indicating the capacity for AuNP to act as both a carrier and adjuvant
Iron	Shen et al. 2012	Fe_3_O_4_	58.7 nm	M BALB/c mouse	OVA	Intravenously	0.2–10 mg/kg	Decreased footpad swelling, infiltration of macro-phages and T-cells, and IFN-y, IL-6, TNF-α levels
	Hsiao et al. 2018	Fe_3_O_4_	58.7 nm	M BALB/c mouse	OVA	Intravenously	1–100 μg	Attenuation in TH17 responses
Silica	Choi et al. 2011	SiO_2_	7nm	CBA/N mouse	HSEM LLNA	Dermal	10–1,000 μg	SiO_2_NP did not induce phototoxicity or skin sensitization
	Hirai et al. 2015	SiO_2_	30 nm	F NC/Nga slc mouse	HDM	Dermal	20 μl @ 12.5mg/mL	Concurrent exposure to allergen and particles resulted in low-level production of allergen-specific IgG subtypes and increased sensitivity to anaphylaxis
	Ostrowski et al. 2014	SiO_2_	55±6nm	M SKH1 mouse	Oxazolone	Dermal	–	Functionalized nanoparticles had no impact on allergic response to oxazolone in an ACD model
	Smulders et al. 2015	SiO_2_	19nm	M BALB/c mouse	DNCB	Dermal	0.4, 4.0, or 40mg/mL×3d	SiO_2_NP exposure prior to sensitization with DNCB did not alter the stimulation index
Silver	Kim et al. 2013	Ag	10nm	M SPF guinea pig	GPMT	Intradermal injection	0.1 mL @ 1:1 (v/v)	No eye or skin irritation or corrosion. 1/20 guinea pigs developed erythema following subcutaneous injection, leading to its classification as a weak skin sensitizer
				M New Zealand White rabbit	–	Occular application	100 mg	No eye irritation effects 1–72 hours after exposure
	Bhol et al. 2005	Ag	<50nm	F BALB/c mouse	DNFB	Dermal	100 mg 1% nanocrystalline	Reductions in ear swelling, erythema, and inflammation were seen after 4 days of treatment with nanopartide-containing cream
	Smulders et al. 2015	Ag	25–85 nm	M BALB/c mouse	DNCB	Dermal	0.4, 4.0, or 40mg/mLx3d	Exposure prior to sensitization with DNCB did not alter the stimulation index
	Zelga et al. 2016	Ag	-	Guinea pig	GPMT	Dermal	2%	AgNP-containing dressings for chronic wounds were tested via GPMT, wherein 2/10 animals developed slight erythema that resolved after 72 hours, leading to classification as a mild sensitizer
	Korani et al. 2011	Ag	<100nm	M Harley Albino guinea pig	-	Dermal	100–10,000 μg/ml	Dose-dependent increase in number of Langerhans cells recruited to skin
Titanium	Parket al. 2011	TiO_2_	<25nm	F CBA/N mouse	LLNA	Dermal	10–1000 μg/mL	TiO_2_ did not induce skin sensitization
				F Hartley Albino guinea pig	-	Dermal	50 μg	TiO_2_ did not induce phototoxicity or acute cutaneous irritation
	Hussain et al. 2012	TiO_2_	12±2nm	M BALB/c mouse	DNCB	Subcutaneous Injection	0.004–0.4 mg/mL	TH2 adjuvancy, increased DNCB dermal sensitizer potency
	Auttachoat et al. 2014	TiO_2_	<25nm	F BCC3F1 mouse	-	Dermal, Subcutaneous Injection	1.25–250 mg/kg	Dermal exposure did not induce auricular lymph node expansion, despite irritancy response at 5% and 10%, no ear swelling, but lymph node cell proliferation resulted following subcutaneous injection
	Smulders et al. 2015	TiO_2_	15nm	M BALB/c mouse	DNCB	Dermal	0.4, 4.0, or 40mg/mL×3d	Exposure to 4.0 mg/mL of TiO_2_ prior to sensitiza-tion with DNCB resulted in increased stimulation index
	Piasecka-Zelga et al. 2015	TiO_2_		New Zealand albino rabbit	Acute Dermal Irritation	Dermal	0.5 g	5 UV-absorbers containing nano-sized particles were assessed for irritation and sensitization potential. Anatase Ti0_2_-containing agent did not induce irritation, but caused mild sensitization
				Dunkin-Hartley guinea pig	GPMT	Dermal	0.1 mL/site	
Zinc	Piasecka-Zelga et al. 2015	ZnO	APS 396 nm, containing nanoparticles	New Zealand albino rabbit	Acute Dermal Irritation	Dermal	0.5 g	5 UV-absorbers containing nano-sized particles were assessed for irritation and sensitization potential. Z11 modifier caused minor dermal irritation and mild sensitization
				Dunkin-Hartley guinea pig	GPMT	Dermal	0.1 mL/site	
	Kim et al. 2016	ZnO	20–50 nm, 13.1 m^2^/g	M Sprague-Dawley Rat, M New Zealand White rabbit, M guinea pig	GPMT	Dermal	50%	ZnONP did not induce dermal sensitization, acute dermal toxicity, irritation, or corrosion

Summary of studies investigating metal nanomaterial immune effects in the skin and select *in vitro* studies using dermal cells, grouped by metal. APS: average particle size, DNCB: dinitrochlorobenzene; GPMT: guinea pig maximization test; HDM: house dust mite; HSEM: Human Skin Equivalent Model; LLNA: Local Lymph Node Assay; OVA: ovalbumin; UV: ultraviolet.

**Table 3.** Summary of major findings from studies comparing the effects of various physicochemical properties of metal nanomaterials on dermal allergy grouped by property of interest.

Property investigated	Author/year	Metal	Study design	Property variations	Findings
Size			Kang H et al. 2017	Ag	RBL-2H3 mast cells F NC/Nga mouse, HDM AD: 40 ng	5, 100 nm AgNP	5 nm AgNP induced increases in ROS levels, intracellular calcium, and granule release in mast cells *in vitro* and earlier and more severe lesions in an AD model *in vivo*
			Nabeshi et al. 2010	Si	XS52 mouse epidermal Langerhans cell, 0.1–1000 ug/mL	70, 300, 1000 nm SiNP	Cellular uptake and cytotoxicity increased with reductions in particle size
			Yoshida et al. 2010	Si	XS52 mouse epidermal Langerhans cell, 0–100 μg/mL	70, 300, 1000 nm SiNP	ROS generation by LC was higher following exposure to the smaller amorphous SiNP
			Hirai et al. 2012b	Si	M NC/Nga mouse HDM ACD Intradermal injection: 250 μg	1136nm SiO_2_NP: 33.2 mV, 264 nm SiO_2_NP: 25.8 mV, 106 nm SiO_2_NP: 24.3 mV, 76 nm SiO_2_NP: 19.5 mV, 39 nm SiO_2_NP: 14.0mV	Reduction in size of SiO_2_NPs caused enhanced IL-18 and TSLP production, leading to an enhanced systemic Th2 response and aggravation of skin lesions following challenge with house dust mite
			Kang S et al. 2017b	Au	Mouse footpad injection OVA	7, 14, 28 nm AuNP	Size-dependent increase in cellular uptake by DC, T-cell cross-priming, and activation.after injection into footpad, higher delivery efficiency to lymph nodes was associated larger NPs
			Yanagisawa et al. 2009	Ti	M NC/Nga mouse HDM Intradermal injection: 20 μg	15 nm TiO_2_NP-110 m^2^/g 50 nm TiO_2_NP-20–25 m^2^/g 100 nm TiO_2_NP-10–15 m^2^/g	TiO_2_NP aggravated AD skin lesions, caused increased IL-4 production, IgE levels, and histamine levels, but decreased IFN-γ expression. Effects were not dependent on size
			lives et al. 2014	Zn	F BALB/c mice, OVA ACD Dermal application: 16.67 mg/mL	20, 240 nm ZnONP	Smaller sized ZnONPs were able to penetrate the skin, whereas larger particles were not. Both particles diminished local skin inflammation, but ZnONPs exhibited higher suppressive effects and increased IgE production
	CRG						
			Jang et al. 2012	Zn	CBA/N Mouse LLNA: 25, 50, or 100 μg/mL ZnONP HSEM EpiDerm skin irritation Draize skin irritation: 50 μg/mL	20 nm, 29 mV or 40 mV ZnONP 100 nm, 24 mV or 29 mV ZnONP	ZnO are not dermal sensitizers and do not induce skin irritation irrespective of size and zeta potential, but may induce phototoxicity
			Jatana et al 2017b	Si	M/F hairless C57BL6 mouse DNFB ACD Dermal application	32.7 nm SiO_2_ nanosphere: 25.4 mV 66.5 nm SiO_2_ nanosphere: 45.7 mV 69.3 nm SiO_2_ nanosphere: 17.7mV 184.9 SiO_2_ nanosphere: 33.5 mV 440.0 nm SiO_2_ nanosphere: 66.0 mV	Small negative and neutral-charged nanoparticles exhibited an immunosuppressive effect, whereas positively-charged particles did not. Positively-charged nanoparticles penetrated skin to a lesser extent. Studies also included lOOnm TiO_2_NP, 20 nm AgNP, and 20 nm AuNP
			Schaeublin et al. 2010	Au	HaCaT human keratinocyte cells 10 μg/mL–25 μg/mL	1.5 nm AuNP Positive, neutral, or negatively-charged	Cell morphology was disrupted by AuNPs of all 3 charges in a dose-dependent manner. Charged AuNPs caused dose-dependent cytotoxicity and mitochondrial stress
	CRY	SA	Lee et al. 2011	Si	J774A.1 mouse macrophages: 0–1,000 ug/mL, 1 or 3 d LLNA: F BALB/c, 1 mg/ ear × 3 tx	100 nm spherical: mesoporous SiO_2_ 1150 m^2^/g colloidal SiO_2_-40 m^2^/g	Higher surface area caused decreased cytotoxic and poptotic cell death. Similarly, higher surface area induced lower expression of pro-inflammatory cytokines. Lower surface area Si particles acted as an immunogenic sensitizer in the LLNA
Size			Maquieira et al. 2012	Al	Mice and rabbits Intradermal injection	40, 3000 nm amorphous AI_2_O_3_, 300 nm crystalline AI_2_O_3_	AINP served as both carrier and adjuvant leading to hapten-specific antibody production dependent on size and crystallinity
			Braydich-Stolle et al. 2009	Ti	HEL-30 mouse keratinocytes 0–150 μg/mL 24 h exposure	100% anatase TiO_2_: 6.3, 10, 40, 50, 100 nm 61% anatase, 39% rutile TiO_2_: 39 nm 40% anatase, 60% rutile TiO_2_: 39 nm 75% anatase, 25% rutile TiO_2_: 26 nm Amorphous TiO_2_: 40 nm 100% rutile TiO_2_: 51 nm	Both size and crystal structure contributed to toxicity in vitro. Smaller size and less agglomeration increased cytotoxicity. 100% anatase TiO_2_ particles, regardless of size, induced cell necrosis, whereas the rutile TiO_2_ nanoparticles initiated apop-tosis through formation of ROS
		MOD	Orlowski et al. 2013	Ag	291.03C mouse keratinocyte 1–10 μg/mL 24 h exposure	Tannic acid-modified AgNP: 13, 33, and 46 nm Unmodified AgNP: 10 −65 nm	Unmodified, but not modified, AgNP increased production of MCP-1 by keratinocytes and upregulation of TNF-α, attributable to increased ROS production
			Li et al. 2016	Si	F C57BL/6 mouse 5mg injection	Unmodified mesoporous SiNP PEG, PEG-RGD, PEG-RDG- modified SiNP	PEG modification significantly enhanced DC activation in vitro and innate immune cell infiltration in vivo. PEG-modification resulted in less recruitment of DC to area of injection

Summary of study design and major findings from studies comparing the effects of various physicochemical properties of metal nanomaterials on dermal allergy grouped by study property of interest. Properties of interest: size, CRY (crystallinity), CRG (surface charge), SA (surface area), and MOD (surface modification). Reported particle size (nm), specific surface area (m2/g), zeta potential (mV), pore volume (cm3/g), in vitro dose concentration (mg/mL). DC: dendritic cell; DNFB: dinitrofluorobenzene; HDM: house dust mite; LC: Langerhans cell; LLNA: Local Lymph Node Assay; HSEM: Human Skin Equivalent Model; OVA: ovalbumin; PEG: poly(ethyleneglycol) modification, PEG-RDG/RGD; ROS: reactive oxygen species; TSLP: thymic stromal lymphopoietin.

**Table 4.** Metal nanomaterials and corresponding physicochemical properties shown to influence immunological processes involved in the development and augmentation of ACD.

AOP Step	Metal nanomaterial effect	Metal	Properties implicated	Source
Sensitization Bioavailability	Increased potential for nanomaterial penetration of intact skin	Au	size, crg	Labouta et al. 2011
	Increased potential for nanomaterial penetration of damaged skin	Ti	size, cry	Monteiro-Riviere et al. 2011
	Increased release of ions from parent nanomaterials	Pd	size	Filon et al. 2016
	Accumulation of nanomaterial in follicles and skin folds	Zn	mod	Leite-Silva et al. 2013
Molecular initating event	Increased potential for metal antigen formation	Ti	size	Vamanu et al. 2008
	Adsorbed protein conformational changes	Au	size	Bastus et al. 2009
Cellular response	Selective uptake by LC	Si	size	Nabeshi et al. 2010
	Direct activation of DC	Ti	size, cry, mor	Schanen et al. 2009
	Release of DAMPs from skin epithelial cells > activation of DC	Fe	size	Murray et al. 2013
	Release of DAMPs from dermal immune cells > activation of DC	Si	size, SA	Lee et al. 2011
	Altered immunogenicity from adsorption of LPS to surface	Au	size, mod, hyd	Li et al. 2017
Organ response	Depot formation > altered delivery kinetics	Si	agg	Hirai et al. 2015
	Nanomaterial antigen vehicle > altered delivery kinetics	Ti	–	Smulders et al. 2015
	Accumulation in DC endocytic compartments > interference with antigen processing	Si	mod, crg	Shahbazi et al. 2014
	Enhanced capacity for cross-presentation to CD8+ T-cells	Fe	crg	Mou et al. 2017
	Increased TH1 signaling	Co		Cho et al. 2012
Elicitation Organism response	Increased permeability of endothelial cells	Al	–	Oesterling et al. 2008
	Increased effector T-cell recruitment	many	–	Lozano-Fernandez et al. 2014
	Increased number of DC for T-cell activation	Si	mod	Li et al. 2016
	Increased number of skin macrophages	Fe	–	Yun et al. 2015
	Increased neutrophil influx to the skin	Ti	–	Goncalves, 2011
	Increased number of skin mast cells	Ag	size	Kang H et al. 2017
	Altered T-cell response to mitogens/allergens	Pd	size, sol	Reale et al. 2011
	Increased IgE-independent mast cell degranulation	many	size, SA, crg	Johnson et al. 2017
Chronic effects	Compromised skin repair mechanisms	Ag	–	Vieira et al. 2017
	Aggravation of allergic lesion severity	Si	size	Hirai et al. 2012b
	Compromised barrier integrity > increased penetration of allergens	Ag	size, sol	Koohi et al. 2011

Adverse Outcome Pathway (AOP) steps in the sensitization and elicitation phases of allergic contact dermatitis, metal nanomaterials shown to impact individual steps and cells involved, and physicochemical properties associated with effects are shown. Physicochemical properties of interest include size, agglomeration (agg), surface modification (mod), surface area (SA), solubility (sol), surface charge (crg), morphology (mor), crystallinity (cry), hydrophobicity (hyd), surface chemistry (SC). ND (not determined) notation in metal column indicates a study demonstrating a critical role for a specific nanomaterial physicochemical property on the cellular event, but was demonstrated using nonmetal nanomaterials. Findings may be applicable to metals, but have not been demonstrated with individual metal nanomaterials.

**Table 5.** Summary of major findings from studies characterizing the effect of metal nanomaterials on respiratory allergy grouped by metal.

Metal	Author/year	Material	Size	Animal or cell type	Mode	Exposure route	Dose	Findings
Aluminum	Braydich-Stolle et al. 2010	Al	48.08±21.0 nm	Human A549 type ∥ pneumocyte U937 alveolar macrophage 3:1 co-culture		*In vitro*	5–500 μg/mL 24 h	Exposure impaired bacterial phagocytic function
		AI_2_O_3_	32.71 ± 28.3 nm					Exposure impaired bacterial phagocytic function, induction of NFκB pathway
Cerium	Park E-J et al. 2009	CeO_2_	130 nm	M ICR mouse	-	Intratracheal Instillation	50, 100, 200, 400 mg/kg	Differentiation of naive T-cells and TH1 cytokine production
	Meldrum et al. 2018	CeO_2_	<25nm APS 166.5 nm agglomerates	F BALB/c mouse	HDM	Intranasal Instillation	75 or 750 μg/kg	Repeated exposure to CeO_2_NP in the presence of HDM caused increased lung eosinophils, mast cells, plasma IgE, IL-4, and goblet cell metaplasia
Cobalt	Cho et al. 2012	Co_3_O_4_	18.4± 5.0 nm	F Wistar rat	-	Intratracheal Instillation	150 cm^2^ SA	Exposure caused pulmonary alveolar proteinosis and TH1/TH17 dominant response
	Verstaelen et al. 2014	CoO	7.1 nm	BEAS-2B, A549 epithelial cells		*In vitro*	1–60 μg/mL	Alterations in expression of genes associated with innate immunity, T-cell activation, and leukocyte adhesion
Copper	Cho et al. 2012	CuO	23.1 ± 7.2 nm	F Wistar rat	-	Intratracheal Instillation	150 cm^2^ SA	No immunoinflammatory reaction
	Park et al. 2015	CuO	<50 nm	F BALB/c mouse	OVA	Intranasal Instillation	25, 50, 100 μg/kg	Increased AHR, IgE and mucus production
	Lai et al. 2018	CuO	46.5 nm	C57BL/6 mouse	-	Intranasal delivery	1, 2.5, 5, 10 mg/kg	Aggravated pulmonary inflammation, collagen accumulation and expression of progressive fibrosis markers in lungs
Iron	Park et al. 2015	Fe_2_O_3_	1013 ± 4.2 nm	M ICR mouse	-	Intratracheal instillation	0.5, 1, or 2 mg/kg	TH1-polarized inflammatory response, GM-CSF, MCP-1, and MIP-1 increase, and increased expression of CD80, CD86, and MHC II expression on lung APCs
	Park et al 2010c	Fe_3_O_4_	5.3 + 3.6 nm	M ICR mouse	-	Intratracheal instillation	250, 500, 1,000 μg/kg	Increases in TH1/TH2 cytokines, B cells, and IgE levels
Gold	Hussain et al. 2011	Au	40 nm	M BALB/c mouse	TDI	Aspiration	40 μL @ 0.8 mg/kg	3x AHR increase
	Baretto et al. 2015	Au	6.3 nm	Swiss Webster and A/J mouse	OVA	Intranasal Instillation	6, 60 μg/kg	Inhibited allergen-induced accumulation of inflammatory cells, pro-inflammatory cytokine production. In A/J mice, AuNPs prevented mucus production and AHR
Nickel	Cho et al. 2012	NiO	5.3 nm	F Wistar rat	-	Intratracheal Instillation	150 cm^2^ SA	Exposure caused pulmonary alveolar proteinosis and TH1/TH17 dominant responses
	Baker et al. 2016	Ni	20 nm	M C57BL/6 WT or T-bet−/− mouse	-	Aspiration	4 mg/kg	Increased airway remodeling in T-bet knockout mice with susceptibility to TH2 responses
	Lee et al. 2016	NiO	5.3 ± 0.4 nm	F Wistar rat	-	Intratracheal Instillation	50, 100, 200 cm^2^	Acute neutrophilic inflammation, and eosinophils recruited at days 3 and 4 via eotaxin release
	Chang et al. 2017	NiO	-	M Wistar rat	-	Intratracheal Instillation	0.015–0.24 mg/kg	Alterations in TH1/TH2 balance were indicative of nitrative stress and NFk-B activation
Platinum	Park et al. 2010b	Pt	20.9 ± 11.4 nm	M ICR mouse	-	Intratracheal Instillation	-	Increase in serum IgE, lung TH2 cytokines, and decrease in CD4/8 ratio
	Onizawa et al. 2009	Pt	2 ± 0.4 nm	DBA/2 mouse	-	Intranasal instillation	x3 d	PtNP exerted protective effects from cigarette smoke, prevented NFk-B activation, and neutrophilic inflammation
Silica	Brandenberger et al. 2013	Si	90 nm	F BALB/c mouse	OVA	Intranasal instillation	0, 10, 100,400 μg	Co-exposure during sensitization caused dose-dependent enhancement of OVA-specific IgE, lung eosinophils, mucus cell metaplasia, and TH2/TH17 cytokine production
Silver	Park H et al. 2010	Ag	6 ± 0.29 nm	F C57BL/6 mouse	OVA	Inhalation	5 × 20 ppm, 40 mg/kg	Decreased AHR, TH2 cytokines, and ROS levels
	Jang et al. 2012	Ag	6.0 ± 0.29 nm	F BALB/c mouse	OVA	Inhalation	20 ppm/40 mg/kg 5× for 24 h	Suppressed mucus production via VEGF signaling alterations
								
	Su et al. 2013	Ag	33 nm	F BALB/c mouse	OVA	Inhalation	3.3 ± 0.7 mg/m^3^ 6h/7 × 7 d	Increased OVA IgE, proteins associated with immune processes were altered
	Chuang et al. 2013	Ag	33 nm	F BALB/c mouse	OVA	Inhalation	3.3 mg/m^3^ 6h/d × 7 d	Increased Penh, recruitment of neutrophils, lymphocytes, and eosinophils to the airways
	Xu et al. 2013	Ag	141 nm	F BALB/c mouse	OVA	IP Injection	0.4, 2, 10 mg/kg	ncreased OVA-IgG and TH2 responses, local activation and recruitment of leukocytes
Titanium	Ahn et al. 2005	TiO_2_	0.29 μm	M Sprague-Dawley rat	-	Intratracheal Instillation	4 mg/kg	Increased BAL IL-13 levels, IL-13-producing mast cells, and goblet cell hyperplasia
	Park et al. 2009	TiO_2_	20 nm	ICR mouse	-	Intratracheal Instillation	5, 20, or 50 mg/kg	Increased BAL and serum IgE levels, altered TH1/TH2 cytokines, increased B cell distribution
	Larsen et al. 2009	TiO_2_	28 nm	F BALB/c mouse	OVA	IP Injection	2–250 μg	TH2 adjuvancy, increased IgE, IgG1, and eosinophil levels
	Rossi et al. 2010	TiO_2_	10 × 40 nm	F BALC/c/Sca mouse	OVA	Inhalation	2 h/d, 3 d/w, x4 w @ 10 mg/m^3^	Allergic pulmonary inflammation suppressed by TiO_2_NP
	Gustafsson et al. 2011	TiO_2_	21 nm	M Dark Agouti rat	-	Intratracheal instillation	5 mg/kg	ncreased eosinophil, DC numbers in lungs, lymphocytes recruited mostly CD4+, also included CD8+ T-cells, B-cells, and CD25+ T-cells
	Hussain et al. 2011	TiO_2_	15 nm	M BALB/c mouse	TDI	Aspiration	40 μL @ 0.8 mg/kg	2× increase AHR
	Scarino et al. 2012	TiO_2_	5 nm APS, 168/171 nm agg.	M Brown Norway rat	OVA	Inhalation	9.4 or 15.7 mg/m^3^	Significantly decreased lung leukocytes and plasma/BAL IL-4, IL-6, and IFN-γ over OVA controls
	Jonasson et al. 2013	TiO_2_	21 nm	F BALB/c mouse	OVA	Inhalation	32 ± 1 μg	Aggravated allergic response dependent on dose and timing
	Fu et al. 2014	TiO_2_	21 nm	M Sprague-Dawley rat	-	Intratracheal Instillation	0.5, 4.0, 32 mg/kg 2x/w × 4 w	Deposition in lymph nodes, increased T and B-cell proliferation following mitogen stimulation, enhanced NK activity in spleen, increased B-cells in the blood
	Choi et al. 2014	TiO_2_	P25	M New Zealand White Rabbit	-	Intratracheal instillation	10, 50, 250 μg	Dose-dependent eosinophil influx and inflammation in the lung, but not neutrophil or lymphocyte influx
	Gustafsson et al. 2014	TiO_2_	21 nm	M Dark Agouti rat M Brown Norway rat	OVA	Inhalation	168–159 μg/d × 10 d	Exposure decreased eosinophilia in OVA-sensitized DA and BN rats, but neutrophils/lymphocyte increase in DA rats
	Mishra et al. 2016	TiO_2_	4–8 nm	BALB/c mouse	OVA	IP Injection	200 μg	Augmented AHR, biochemical markers of damage, and induced a mixed TH1/TH2 response
	Kim et al. 2017	TiO_2_	75 nm	F BALB/c mouse	OVA	Inhalation	50 μg/m^3^ × 3 d	Exposure exacerbated AHR and inflammation, increases in IL-1, IL-18
Zinc	Roy et al 2014b	ZnO	<50nm	F BALB/c mouse	OVA	IP Injection	0.25, 0.5, 1, 3 mg	Administration with OVA caused increased OVA-IgG1, IgE, eosinophil, and mast cell numbers in lungs and spleen
	Roy et al 2014a	ZnO	<50nm	F BALB/c mouse	OVA	IP Injection	1, 2, 4, and 12 mg/mL	Adjuvant effect on OVA allergy by signaling through TLRs and Src kinase leading to inflammatory responses
	Huang et al. 2015	ZnO	181.5 nm low dose 360.0 nm high dose	F BALB/c mouse	OVA	Aspiration	0.1/0.5 mg/kg	Exposure simultaneous to OVA sensitization resulted in eosinophil recruitment and TH2 adjuvancy

Summary of findings from *in vivo* studies investigating immune effects of metal nanomaterials in the lung and select *in vitro* studies in pulmonary cells, grouped by metal. AHR: airway hyperreactivity; APC: antigen-presenting cell; APS: average particle size; DC: dendritic cell; HDM: house dust mite; IP: intraperitoneal; LPS: lipopolysaccharide; MHC: major histocompatibility complex; OVA: ovalbumin; ROS: reactive oxygen species; RSV: respiratory syncytial virus; TLR: Toll-like receptor; WT: wild-type.

**Table 6.** Summary of major findings from studies comparing the effects of various physicochemical properties of metal nanomaterials on respiratory allergy grouped by property of interest.

Property investigated		Author/year		Metal		Study design	Property variations			Findings
Size		de Haar et al. 2006		Ti		F BALB/cANNCrl mouse	Fine TiO_2_: 250 nm, 6.6 m^2^/g			Exposure to equal mass doses of fine and ultrafine TiO_2_ resulted in
						Ovalbumin, 200 μg intranasal	Ultrafine TiO_2_: 29.0 nm, 49.8 m^2^/g			increased TH2 cytokines and serum OVA-specific IgE and IgGI only
										in animals exposed to ultrafine TiO_2_
		Yoshida et al. 2011		Si		F BALB/c mouse, Ovalbumin	Amorphous silica			Smaller particles induced higher levels of OVA-specific IgE, IgG, and
						Intranasal: 10, 50, or 250 μg/ mouse x3	30, 70, 300, or 1000 nm			IgG1. Splenocytes from mice exposed to the smallest particle pro-
										duced higher levels of TH2 cytokines than other groups.
		Liu et al. 2010		Ti		Rats, Intratracheal instillation	5 or 200 nm TiO_2_			Decreased chemotactic ability, expression of Fc receptors/MHC II by
						0.5, 5, or 50 μg/mL				alveolar macrophages. Phagocytic function was increased at low
										doses and decreased at high doses
		Chang et al. 2014		Ti		M Sprague Dawley rat	21 nm TiO_2_NP: 80% anatase, 20% rutile,			Increased macrophage accumulation and alteration of TH1/TH2 status
						Intratracheal instillation: x2, x4 w	1–2 μm TiO_2_: anatase			
						0.5, 4, 32 mg/kg				
		Ban et al. 2013		Fe		F BALB/c mouse, Ovalbumin	Submicron Fe_2_O_3_: 147±48nm, 6 m^2^/g			High and medium doses of both Fe particles caused decreases in
						Intratracheal instillation:	Fe_2_O_3_NP: 35±14nm, 39 m^2^/g			eosinophil influx and OVA-specific IgE levels. However, at the low
						4x (100, 250, or 500 μg/mouse)				dose, submicron particles had no effect on allergy, whereas nano-
										particles had an adjuvant effect on the TH2 response to OVA
SA		Rossi et al. 2010		Ti		F BALB/c/Sca mouse, Ovalbumin	Rutile TiO_2_NP: <5 μm, 2 m^2^/g			Allergic pulmonary inflammation was dramatically suppressed in asth-
						Inhalation: 10±2mg/m^3^ × 12	Rutile TiO_2_NP: 10 × 40 nm, 132 m^2^/g			matic mice exposed to either size TiO_2_. Leukocyte number, cyto-
										kines, chemokines, and antibodies were significantly decreased.
		Parketal. 2015		Si		F BALB/c mouse, Ovalbumin	Spherical SiNP: 12.7 m^2^/g			Acute SiNP exposure induced significant airway inflammation and
						Intranasal inoculation	Mesoporous SiNP: 70.6 m^2^/g			AHR. Spherical SiNPs induced the greatest degree of exacerbation
							PEGylated SiNP: 12.7 m^2^/g			of allergic effects in the OVA model
		Han et al. 2016		Si		F BALB/c mouse	Spherical SiNP: 12.7 m^2^/g, 119.6nm			Sensitized mice exposed to S-SiNP and M-SiNP exhibited elevated
						Ovalbumin	Mesoporous SiNP: 70.6 m^2^/g, 100.5 nm			AHR over controls. M-SiNPs induced the greatest degree adjuvan-
						Intranasal inoculation: 10 mg/kg 6x	PEGylated SiNP: 12.7 m^2^/g, 439.1 nm			ticity, whereas PEG-SiNP caused the least toxic effects
MOD		CRG Seydoux et al. 2016		Au		F BALB/c mouse	90 nm AuNP:			APCs preferentially took up cationic AuNPs, causing upregulation of
						AuNP intranasal instillation: 10 μg	NH_2_-PVA, 7.2 mV			co-stimulatory molecules. Positive AuNPs enhanced OVA-specific
							COOH-PVA 8.2 mV			CD4+ T-cell stimulation in the lung draining lymph nodes
		Marzaioli et al. 2014		Si		F BALB/c mouse, Ovalbumin	Amorphous SiO_2_NP 15 nm:			Uncoated SiO_2_NPs induced proinflammatory and immunomodulatory
						Intratracheal instillation: 50 μg	Uncoated 38 mV, PEGylated 26 mV,			effects with increases in lung inflammatory cells, TH2 cytokines.
							Phosphate-coated 43 mV, Amino-coated 0 mV			Amino and phosphate surface modifications mitigated these
										effects, whereas PEG coating did not.
		Omlor et al. 2017		Au		F BALB/c mouse, Ovalbumin	5 nm AuNP, PEGylated or citrated			Asthmatic condition increased nanoparticle uptake. Systemic uptake is
						Intranasal instillation				higher for PEGylated AuNP compared to citrated AuNPs, but both
										inhibited inflammatory infiltrates and AHR, wherein inhibition was
										more significant following exposure to citrated AuNPS
		Vennemann		Zr		F Wistar rat	APTS, TODS, PGA, or acrylic acid coated			Surface coating had minimal effects on inflammation in the lungs of
		et al. 2017				Intratracheal instillation	9–1 Onm ZrO_2_NPs			rats, but had significant effects on allergic response.
Size MOD		Alessandrini		Ag		F BALB/c mice, Ovalbumin	PVP-coated AgNP: 97 nm, 6.2 m^2^/g, 7mV			Ag50-PVP significantly reduced OVA-induced inflammatory infiltrate in
		et al. 2017				Intratracheal instillation: 1–50 μg	PVP-coated AgNP: 134nm, 4.5 m^2^/g, 7mV			sensitized mice. Lung microbiome was altered dependent
							Citrate-AgNP: 20 nm, 30 m^2^/g, 45 mV			on coating.
		Seiffert et al. 2015		Ag		Brown Norway and Sprague-Dawley rats	PVP-coated AgNP: 20 or iiOnm			Smaller AgNPs increased AHR on d 1, which persisted to d 7 for the
						Intratracheal instillation: 0.1 mg/kg	Citrate-capped AgNP: 20 or iiOnm			citrate AgNPs only. 20 nm AgNP was more pro-inflammatory but
										little difference between different surface coatings
		CRY Horie et al. 2015		Zn		F C57BL/6N mouse, Ovalbumin	Rutile TiO_2_, AI(OH)_3_ surf: 30–50 nm 37.1 m^2^/g			Serum total and OVA IgE, IgG1 increased in mice treated with the
				Ti		Pharyngeal aspiration: 50 μg	ZnO: 21 nm, 49.6 m^2^/g			uncoated ZnO particle. However, ZnCI_2_ did not produce similar
				Si			ZnO, SiO_2_ Surface: 25 nm, unknown SA, ZnCI_2_			exacerbations. TiO_2_ and SiO_2_ did not affect OVA-lgE or IgG levels.
							Amorphous SiO_2_: 7nm 300 m^2^/g, 34 nm 80 m^2^/g			
SA	Sandberg		Si		LPS-primed RAW264.7 mouse macrophages, pri-			64 nm Si, 650cm^2^/mg	Non-crystalline SiO_2_ particles in both nano and micron size ranges	
	et al. 2012				mary rat lung macrophages			369 nm Si, 90 cm^2^/mg	induced IL-1β release from LPS-primed macrophages following	
					0, 50, 100, 250, 500 μg/mL, 6h			~20nm Fumed Si (aerosol), 1880cm^2^/mg 500nm-10 μm Fused Si (suprasil), 23cm^2^/mg	uptake, phagosomal leakage, and activation of the NALP3 inflam-	
									masome. Particle surface area, reactivity, and uptake all influenced	
									the degree of mediator release by cells	
	Vandebriel		Ti		F BALB/c mouse, Ovalbumin			Uncoated TiO_2_NP:	Rutile TiO_2_NP caused the greatest increase in OVA-specific serum IgE	
	et al. 2018				Intranasal exposure: 120 μg TiO_2_			10–30 nm rutile or 10–25 nm anatase	and IgGI. Neutrophils recruited by rutile, but not anatase	
	SOL Jeong et al. 2015		Co		F Rat, Intratracheal Instillation			CoO: 65.4± 2.8 nm, 92.65% solubility	Soluble CoNP induced eosinophilic inflammation, whereas insoluble	
					80, 200, 800 μg/mL @ 0.5 mL			Co_3_O_4_: 20.2 ± 0.4 nm, 11.46% solubility	CoONP induced neutrophilic inflammation	
	Cho et al. 2011		Zn		F Wistar rat			10.7±0.7nm ZnONP 50 or 150cm^2^/rat	ZnONP induced eosinophilia, proliferation of airway epithelial cells,	
					Intratracheal instillation			Zn^2+^ ions 92.5 μg/rat	goblet cell hyperplasia, and increased IgE levels, and decreased	
									IgA- findings which were also seen following instillation of Zn ions	

Summary of study design and major findings from studies comparing the effects of various physicochemical properties of metal nanomaterials on respiratory allergy grouped by study property of interest. Properties of interest: size, CRY (crystallinity), MOR (morphology), MOD (surface modification), CRG (surface charge), and SA (surface area), and SOL (solubility). Reported particle size (nm), specific surface area (m2/g), zeta potential (mV), pore volume (cm3/g), in vitro dose concentration (mg/mL). AHR: airway hyperreactivity; APC: antigen-presenting cell; APTS: aminopropylsilane modification; DC-SIGN: dendritic cell-specific intercellular adhesion molecule-3-grabbing non-integrin; MDDC: monocyte-derived dendritic cell; MHC: major histocompatibility complex; OVA: ovalbumin; PDI: polydispersity index; PEG: poly(ethylene glycol) modification; PGA: poly(lactic-coglycolicacid) modification; PVA: PVP polyvinylpyrrolidone modification; ROS: reactive oxygen species; TODS: tetraoxidecanoic acid modification

**Table 7.** Metal nanomaterials and corresponding physicochemical properties shown to influence immunological processes involved in the development and augmentation of asthma.

AOP step	Metal nanomaterial effect	Metal	Properties implicated	Source
Sensitization Bioavailability	Increased potential for inhalation	many	dustiness	Evans et al. 2003
	Evasion of uptake by pulmonary macrophages	Si	size, crg, mod	Oh et al. 2010
	Evasion of entrapment by pulmonary mucus	ND	size, crg	Murgia et al. 2016
	Prolonged retention in airways	Al	spec, mor	Park et al. 2017
	Direct translocation across lung epithelial tissue to lymphatics	Au	size, crg	Kreyling et al. 2014
Molecular initating event	Increased potential for metal antigen formation	Ti	size	Vamanu et al. 2008
	Increased protease activity of protein allergens	Au	–	Radauer-Preiml et al. 2016
Cellular response	Adsorption of LPS to nanomaterial surface	Au	size, hyd, mod	Li et al. 2017
	Increased recruitment of DC to lung	Al	mod	Li et al. 2010
	Direct activation of DC	Ti	size, cry	Winter et al. 2011
	Release of DAMPs from immune cells > activation of DC	Zn	size, mor, SA	Hsaio et al. 2011
	Release of DAMPs from epithelial cells > activation of DC	Ag	size, mod, SA, sol	Hamilton et al. 2014
Organ response	Increased CD4^þ^ T-cell presentation efficiency	Ti	size, mor, cry	Schanen et al. 2009
	Increased polarization of CD4^þ^ T-cells to T_H_2 phenotype	Si	size, mod, SA	Vallhov et al. 2012
	Increased number of B-cells	Ti	–	Park et al. 2009
	Alteration in B-cell expansion/maturation	Au	size	Lee et al. 2014
	Increased production of total IgE	Pt	–	Park et al. 2010b
	Increased production of allergen-specific IgE	Zn	size, sol, cry	Horie et al. 2015
Elicitation				
Organism response-early phase reaction	Increased IgE-dependent mast cell degranulation	Au	size, mod	Huang et al. 2009
	Increased IgE-independent mast cell degranulation	many	size, SA, crg	Johnson et al. 2017
	Increased number of lung mast cells	Ce	–	Meldrum et al. 2018
	Altered mast cell exocytic function and granule release	Si	SA	Maurer-Jones et al. 2010
	Altered expression of Fc receptors on immune cells	Ti	size	Liu et al. 2010
Organism response- late phase reaction	Increased endothelial adhesion molecule expression	Al	–	Oesterling et al. 2008
	Increased neutrophil recruitment	Ag	size, sol	Arai et al. 2015
	Increased eosinophil recruitment	Co	sol	Jeong et al. 2015
	Increased lymphocyte recruitment	Zr	mod	Vennemann et al. 2017
	Increased AHR	Ag	size, mod	Seiffert et al. 2015
	Increased airway smooth muscle contractility	Co/Fe	–	Kapilevich et al. 2012
	Mucus cell metaplasia/muus hyeprsecretion	Ti	–	Chen et al. 2011
Chronic effects	Epithelial cell proliferation	Zn	sol	Cho et al. 2011
	Increased fibroblast MMP activity extracellular matrix remodeling	Ti	size, cry, mor	Armand et al. 2012
	Myofibroblast accumulation	Cu	–	Lai et al. 2018

Adverse Outcome Pathway (AOP) steps involved in the sensitization and elicitation phases of asthma, metal nanomaterials shown to impact individual steps, and physicochemical properties associated with effects are shown. Physicochemical properties of interest include size, metal speciation (spec), agglomeration (agg), surface modification (mod), surface area (SA), solubility (sol), surface charge (crg), morphology (mor), crystallinity (cry), and hydrophobicity (hyd). ND (not determined) notation in metal column indicates a study demonstrating a critical role for a specific nanomaterial physicochemical property on the cellular event, but was demonstrated using nonmetal nanomaterials. Findings may be applicable to metals, but have not been demonstrated with individual metal nanomaterials.
